# Spectrum and Classification of CFTR and ADGRG2 Variants in Chinese Patients With Isolated CAVD: A Large Cohort Study and Risk Assessment of CFTR Variant Carriage in Couples

**DOI:** 10.1155/humu/5588277

**Published:** 2026-05-25

**Authors:** Ping Yuan, Zhongkun Liang, Ling Zhou, Xiaohui Ji, Shengran Wang, Jing Zhang, Jin Li, Shuoshuo Xie, Yingshi Li, Tuerxunayi Yalikun, Qipeng Sun, Hui Chen

**Affiliations:** ^1^ IVF Center, Department of Obstetrics and Gynecology, Sun Yat-Sen Memorial Hospital, Sun Yat-Sen University, Guangzhou, China, sysu.edu.cn; ^2^ Guangdong Provincial Clinical Research Center for Obstetrical and Gynecological Diseases, Guangzhou, China; ^3^ The Department of Obstetrics and Gynecology, The Third Affiliated Hospital of Guangzhou Medical University, Guangzhou, China, gzhmc.edu.cn; ^4^ The Reproductive Medicine Research Center, The Sixth Affiliated Hospital, Sun Yat-Sen University, Guangzhou, China, sysu.edu.cn; ^5^ IVF Center, The First People′s Hospital of Kashi Prefecture, Kashi, China; ^6^ Department of Organ Transplantation, The Affiliated Guangdong Second Provincial General Hospital of Jinan University, Guangzhou, China

**Keywords:** ADGRG2, carrier risk, CAVD, CFTR, genetic variants, genotype–phenotype correlation

## Abstract

Congenital absence of the vas deferens (CAVD) is a major cause of obstructive azoospermia and male infertility, with its genetic etiology primarily associated with CFTR (autosomal recessive) and ADGRG2 (X‐linked) mutations. However, the genetic spectrum and classification of variants in isolated congenital absence of the vas deferens (iCAVD), as well as the risk of CFTR variant carriage in affected couples, remain incompletely understood. In this cross‐sectional study, we enrolled 199 Chinese iCAVD patients and 148 female partners between 2012 and 2024. CFTR and ADGRG2 variants were identified in 74.87% of iCAVD patients, with CFTR being the predominant pathogenic gene. Notably, 10.14% of couples carried shared pathogenic or likely pathogenic CFTR variants, highlighting the potential reproductive risks. The most common pathogenic variants were CFTR c.1210‐12T (Yu et al., 2012) (5T) and c.4056G > C (p.Gln1352His), whereas c.1666A > G (p.Ile556Val) was classified as likely benign. The c.4056G > C variant exhibited significant regional ethnic characteristics. Furthermore, genotype–phenotype correlation analysis revealed significant differences in semen volume, pH, and fructose levels among different variant subgroups in CBAVD patients. Collectively, these findings provide a comprehensive overview of the genotype–phenotype landscape in a large iCAVD cohort, emphasizing variant classification and reproductive risks associated with CFTR and ADGRG2. This study offers valuable insights for genetic counseling and reproductive planning in affected couples.

## 1. Introduction

Congenital absence of the vas deferens (CAVD) is a leading cause of obstructive azoospermia, accounting for 1%–2% of infertility cases among men [1]. CAVD may manifest as an isolated finding in infertile males (often termed “isolated congenital absence of the vas deferens [iCAVD]”) or as one of the clinical features of cystic fibrosis (CF) [2, 3]. Defined by the absence of the vas deferens, CAVD is generally subdivided into congenital bilateral absence of the vas deferens (CBAVD) and congenital unilateral absence of the vas deferens (CUAVD). Variants in the CFTR and ADGRG2 genes represent the primary known genetic contributors to both phenotypes [3].

Variants in the CFTR gene (OMIM 602421), which follows an autosomal recessive inheritance pattern, are implicated in the majority of CAVD cases, accounting for approximately 70%–80% of iCAVD [1, 4]. However, the ADGRG2 gene (OMIM 300572), inherited in an X‐linked manner, contributes to about 2% of CAVD cases [3]. Moreover, genotype–phenotype correlations for CFTR‐related disorders can vary significantly across different ethnicities [1]. As a result, the pathogenicity of many iCAVD variants remains poorly understood, even in databases such as CFTR2 (CFTR2.org) and ClinVar (www.ncbi.nlm.nih.gov/clinvar), highlighting the need for further study [5].

In Caucasian populations, the most common CFTR variants associated with CAVD are c.1521_1523delCTT (p.Phe508del), c.1210‐12T [5] (5T), and c.350G > A (p.Arg117His) [1]. By contrast, our previous small‐sample study found that 5T, c.1666A > G (p.Ile556Val), and c.4056G > C (p.Gln1352His) were the most frequent variants in Chinese CAVD patients [4]. The 5T allele, classified as pathogenic in ClinVar database, impairs normal splicing by promoting Exon 10 skipping, thereby significantly reducing levels of normal CFTR mRNA [6]. Notably, c.1666A > G and c.4056G > C have conflicting pathogenicity classifications in the ClinVar database, complicating risk assessment for genetic counseling. This challenge is further exacerbated by variants such as c.579 + 4 T > C and c.1210 − 6 T > A, which are predicted to affect splicing but remain unvalidated in functional studies [4]. In fact, most patients with iCAVD have minimal phenotypes aside from infertility and often lack detailed family histories. Consequently, data on whether both partners in a couple carry the same CFTR variant are scarce, making it difficult to assess the risk of offspring developing CF, given the uncertain severity of these mutations. To date, there have been no reports describing the risk of CFTR variant carriage in CAVD couples.

In this study, we analyzed all exons of the CFTR and ADGRG2 genes in 199 iCAVD patients (including both CBAVD and CUAVD) and performed CFTR genotyping in 148 female partners. We also evaluated the splicing effects of variants with conflicting classifications. To further assess pathogenicity, we compared allele frequencies of the five most common variants in our cohort with data from the Genome Aggregation Database (gnomAD) database (v2.1.1) and conducted family‐member validation in 13 couples. Finally, we investigated genotype–phenotype associations in the iCAVD patients, highlighting that the observed triad of features is not pathognomonic but serves as a critical biological warning sign.

## 2. Materials and Methods

### 2.1. Study Population

This cross‐sectional study enrolled 199 Chinese patients diagnosed with iCAVD and 148 female partners at Sun Yat‐Sen Memorial Hospital, Sun Yat‐Sen University, between 2012 and 2024 (Table S1). In addition, the parents of 13 patients from the cohort of 199 males were recruited for familial cosegregation analysis of the identified variants. Diagnosis criteria included semen analysis showing azoospermia after centrifugation at 3000 g for 15 min on three separate occasions per WHO standards [7, 8], normal follicle‐stimulating hormone (FSH) levels, physical palpation, scrotal ultrasound confirming bilateral epididymis and vas deferens absence, abdominal and transrectal ultrasound verifying seminal vesicles and pelvic vas deferens (if unilateral or bilateral absence was suggested on scrotal ultrasound, transrectal ultrasound was performed to evaluate the seminal vesicles and pelvic vas deferens), normal karyotype, and absence of AZF microdeletions. Exclusion criteria encompassed history of infection, urinary system malformation, cryptorchidism, surgery, toxin/heat exposure, and retrograde ejaculation.

Participants were categorized into three groups based on CFTR and ADGRG2 gene variants. Each patient underwent a comprehensive evaluation by an andrology specialist, including medical history, physical examination with testicular volume measurement, semen volume and pH, seminal plasma biochemical markers (fructose and alpha‐glucosidase), hormone levels (FSH, luteinizing hormone, and inhibin B [INHB]), and genetic testing (chromosomal karyotyping and Y chromosome AZF analysis). Testicular biopsies and pathological examinations were performed to assess spermatogenic function. None exhibited CF or related symptoms or a family history of CF.

### 2.2. DNA Sequence Analysis

Genomic DNA was extracted from peripheral blood samples using a Qiagen commercial kit (Germany). The CFTR and ADGRG2 gene sequences were obtained from the UCSC Genome Browser (Human February 2009Assembly, hg19; http://genome.ucsc.edu). Polymerase chain reaction (PCR) was employed to amplify all coding regions and adjacent intronic regions of the CFTR and ADGRG2 genes using the patients′ genomic DNA. Specific primers designed with Oligo 6.0 (http://www.oligo.net/downloads.html) were utilized for amplification. All exons and their surrounding intronic regions were amplified using targeted PCR primers and subsequently subjected to Sanger sequencing. For family members, the identified variants were confirmed by site‐specific Sanger sequencing. Mutations were annotated based on the GenBank complementary DNA (cDNA) references NM_000492 (CFTR) and NM_001079858 (ADGRG2), with the +1 position corresponding to the adenine (A) of the ATG start codon [9]. Variants were classified in accordance with the 2015 American College of Medical Genetics and Genomics (ACMG) standards and guidelines [10].

#### 2.2.1. In Silico Analysis of CFTR and ADGRG2 Novel Variants

The effects of missense variants were evaluated using computational tools REVEL (https://sites.google.com/site/revelgenomics/about), CADD (https://cadd.gs.washington.edu/), Mutation taster (https://www.genecascade.org/MutationTaster2021/#transcript), PolyPhen‐2 (http://genetics.bwh.harvard.edu/pph2/), and SpliceAI (https://spliceailookup.broadinstitute.org/). Allele frequencies were obtained from the gnomAD (http://gnomad.broadinstitute.org/), with additional variant information gathered from dbSNP (https://www.ncbi.nlm.nih.gov/snp/), ClinVar (https://www.ncbi.nlm.nih.gov/clinvar/), CFTR2 (http://www.cftr2.org/), and HGMD (https://www.hgmd.cf.ac.uk/ac/index.php). The amino acid sequences of novel missense variants for human CFTR (UniProt ID P13569) and ADGRG2 (UniProt ID Q8IZP9) were aligned with those from five species (Pan troglodytes, Macaca mulatta, Rattus norvegicus, and Mus musculus) using CLUSTAL X Version 1.81 [11]. Information on motifs and domains of the wild‐type (WT) CFTR and ADGRG2 proteins was retrieved from UniProt (http://www.uniprot.org/) and InterPro (http://www.ebi.ac.uk/interpro/). Evolutionary conservation of the mutated amino acid residues was analyzed using SnapGene software. Three‐dimensional (3D) structural models of both WT and mutant (MUT) proteins were generated via the AlphaFold Server [12] and visualized using the PyMOL Molecular Graphics System, Version 3.0 (Schrödinger, LLC). Five models were produced, and the structure with the highest predicted template modeling (pTM) score was selected for further analysis.

### 2.3. Minigene Assay

To evaluate the effects of CFTR variants, a minigene system was employed by constructing plasmids containing two to three exons along with the surrounding intronic regions encompassing the variants. Genomic DNA was used to amplify the target DNA fragments, which were then cloned into the pMini‐CopGFP minigene vector (Hitrobio Tech, China) using the ClonExpress II One Step Cloning Kit (Vazyme, Nanjing, China). The constructed minigene plasmids were transfected into HEK293T cells via Lipofectamine 3000 (Invitrogen, United States). After 48 h, total RNA was extracted from the transfected HEK293T cells using TRIzol reagent (Ambion, United States). cDNA was synthesized with the HiScript III RT SuperMix (+ gDNA wiper) (Vazyme, China). Subsequent analyses included PCR, agarose gel electrophoresis, and Sanger sequencing. The specific PCR primers used are listed in Table S2.

### 2.4. Statistical Analysis

Statistical analyses were conducted using R software (Version 4.3.1). A total of 245 variants in the CFTR and ADGRG2 genes were identified among 199 patients with iCAVD. The most prevalent mutations were determined based on allele frequencies. Data visualization and manipulation were performed using the R packages tidyverse, ggsankey, ggalluvial, and ComplexHeatmap. For variables with non‐normal distributions, missing values were imputed using the median of each respective group. Continuous variables are presented as medians with interquartile ranges, whereas categorical variables are expressed as frequencies and percentages. The Kruskal–Wallis test was utilized to assess differences across multiple groups for continuous variables, followed by the Dwass–Steel–Critchlow–Fligner (DSCF) test for post hoc pairwise comparisons. Categorical variables were compared using the chi‐square test. All statistical tests were two‐sided, with significance set at p < 0.05.

## 3. Results

### 3.1. Characteristics of CFTR and ADGRG2 Variants in iCAVD Patients

A total of 63 distinct variants were identified in the CFTR and ADGRG2 genes (Figure 1a and Table S1). Among these, 59 variants were found in the CFTR gene and 4 in the ADGRG2 gene (Figure 1a). Of the 63 variants, 40 were classified as pathogenic or likely pathogenic, 19 as variants of uncertain significance, and 4 as likely benign (Figure 1b and Table S1). Excluding the likely benign variants, the remaining 59 variants were categorized into seven types: missense, nonsense, splicing, frameshift, 5T (c.1210‐12T [5]), 6T (c.1210_12T [6]), and 5 ^′^UTR variants (Figure 1b). Among these, 5T and missense variants were the most common, accounting for 36.70% and 26.63%, respectively (Figure 1b).

Figure 1Variant profiles of CFTR and ADGRG2 genes. (a) A total of 63 variants were identified in the CFTR and ADGRG2 genes from 199 patients. The left column represents the gene associated with each variant, with each square corresponding to an individual patient. The right column shows the frequency of each variant. (b) Classification and distribution of CFTR and ADGRG2 variants by type. (c) CFTR variant status in 148 couples.

**Figure:**
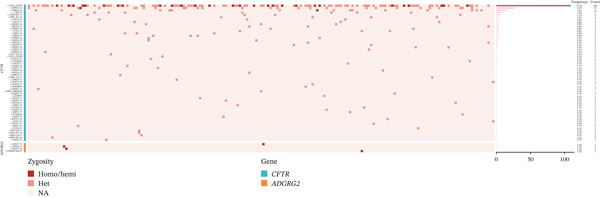
(a)

**Figure:**
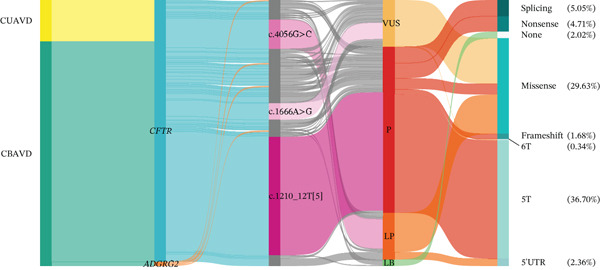
(b)

**Figure:**
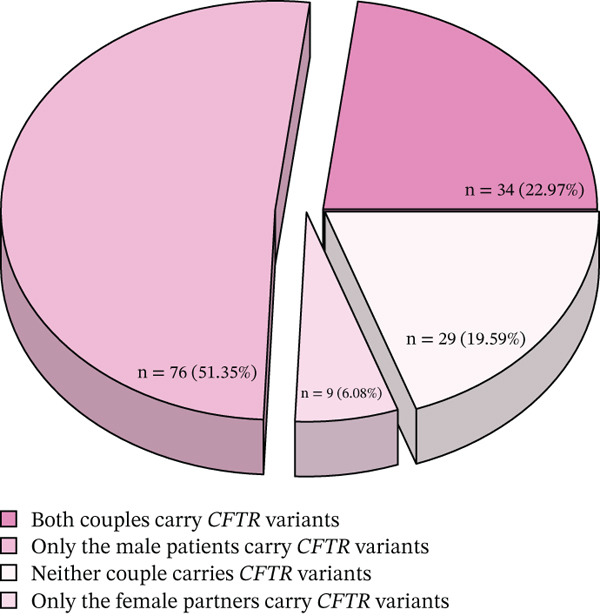
(c)

The most frequent variant identified was 5T, present in 34.42% (137/398) of the alleles among 109 patients, followed by c.4056G > C (p.Gln1352His) in 7.04% (28/398) of alleles from 27 patients, and c.1666A > G (p.Ile556Val) in 4.02% (16/398) of alleles from 15 patients (Figure 1a,b and Table 1), consistent with findings from our previous study in a smaller cohort [4]. Homozygous variants identified included 5T, c.4056G > C, and c.1666A > G, found in 28, 1, and 1 patient(s), respectively (Figure 1a and Table 1).

**Table 1:** Allele frequencies of the five most common variants in CAVD patients compared with gnomAD controls.

Variants	Controls in gnomeAD^a^			CAVD patients in our study				Chisq value	*p*values	OR^c^
	Allele count	Allele number	Allele frequency	Wild type	Heterozygote	Homozygote	Allele frequency			
c.1210_12T[5]^b^	11	9314	1.181 × 10^−3^	90	81	28	0.344 (137/398)	2993.07	< 0.0001	443.93
c.4056G > C	123	10076	1.221 × 10^−2^	172	26	1	0.070 (28/398)	91.10	< 0.0001	6.12
c.1666A > G	460	10086	4.561 × 10^−2^	184	14	1	0.040 (16/398)	0.26	0.6113	0.88
c.−34C > T	0	9034	0.000	192	7	0	0.018 (7/398)	—	< 0.0001	—
c.1766 + 5G > T	4	10074	3.971 × 10^−4^	194	5	0	0.013 (5/398)	—	< 0.0001	13.41

^a^Allele count and frequency in East Asian populations in the genome aggregation database (gnomAD v2.1.1) for XY chromosomes.

^b^The variant c.1210_12T [5] (commonly known as the 5T allele) is equivalent to c.1210‐7_1210‐6del according to HGVS nomenclature.

^c^The OR value is defined as the ratio of odds in the diseased group to that in the control group in this study.

To assess the pathogenic risk of common variants, we compared the prevalence of the five most frequent variants in our patient cohort with their frequencies in the male East Asian population from the gnomAD (v2.1.1). Our analysis revealed that the prevalence of c.1210‐12T [5] (5T), c.4056G > C, c.−34C > T, and c.1766 + 5G > T was significantly higher in our patient cohort compared with controls (p < 0.0001) (Table 1). However, the c.1666A > G variant did not show a significant difference between the case and control groups (Table 1).

### 3.2. Determining the Risk of CFTR Variant Carriage in High‐Risk Couples

A total of 148 couples underwent CFTR gene screening. Of these, 22.97% (34/148) of couples had CFTR variants in both partners. Among the entire cohort, 10.14% (15/148) of couples had pathogenic/likely pathogenic (P/LP) CFTR variants in both partners (Figure 1c). In 51.35% (76/148) of the couples, only the male partner (iCAVD patient) carried a CFTR variant, whereas in 6.08% (9/148), only the female partner carried a variant (Figure 1c). Additionally, 19.59% (29/148) of the couples did not carry any CFTR variants (Figure 1c).

Among the 148 couples, familial cosegregation analysis was performed in 13 families (Table S3). Except for c.1666A > G, which was in cis with c.1680 − 1G > A (classified as pathogenic), all other variants were in trans, including two common variants (5T and c.4056G > C) (Table S3). Given that the allele frequency of the c.1666A > G variant did not significantly differ from controls (Table 1), c.1666A > G was interpreted as a likely benign variant.

### 3.3. Novel Variants Assessment in CFTR and ADGRG2 Genes

Six novel variants were identified: c.373dupA (p.Ile125Asnfs∗34), c.433C > G (p.Leu145Val), c.1342delA (p.Ile448∗), and c.2904A > C (p.Lys968Asn) in CFTR, and c.959delCinsTT (p.Pro320Leufs∗4) and c.1402C > T (p.Gln468∗) in ADGRG2 (Figure 2a and Table S1). All four CFTR variants were heterozygous, and both ADGRG2 variants were hemizygous. These variants were not found in the gnomAD database. Protein sequence alignment showed that two novel missense variants affected highly conserved sites across five species (Figure 2b).

Figure 2Novel variants identified in patients with iCAVD. (a): Sanger sequencing results of novel variants in CFTR and ADGRG2 genes. Arrows indicate variant locations. (b) Alignment of CFTR amino acid sequences across five species: Homo sapiens, Pan troglodytes, Macaca mulatta, Rattus norvegicus, and Mus musculus. (c) Locations of variants in CFTR and ADGRG2 proteins. Upper panel: The wild‐type CFTR protein consists of 1480 amino acids and includes two transmembrane ATP‐binding cassette (ABC) domains (ABC transmembrane Type 1), two ABC transporter nucleotide‐binding domains (ABC transporter), a disordered R region domain, and a PDZ‐binding motif. Bottom panel: The wild‐type ADGRG2 protein consists of 1017 amino acids and contains a G‐protein–coupled receptor (GPCR) domain belonging to class B GPCRs. aa = amino acid. (d) Protein conformation predictions for CFTR variants. Yellow dashed lines represent hydrogen bonds. WT = wild type; MUT = mutation.

**Figure:**
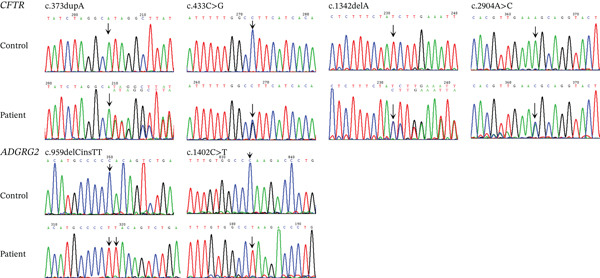
(a)

**Figure:**
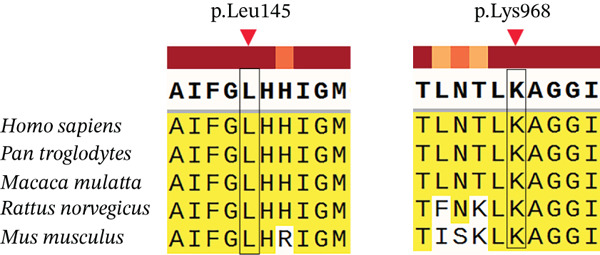
(b)

**Figure:**
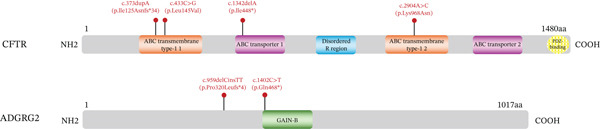
(c)

**Figure:**
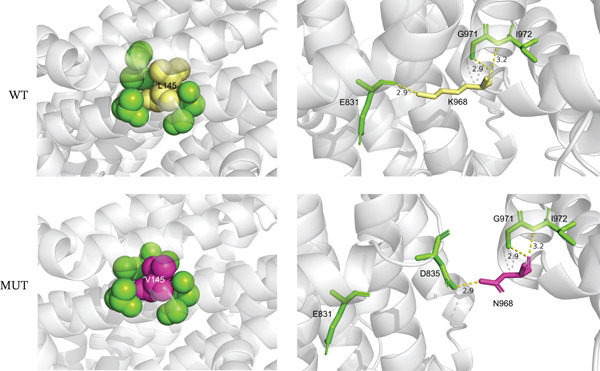
(d)

The c.373dupA (p.Ile125Asnfs∗34) and c.1342delA (p.Ile448∗) variants in CFTR were located in the ABC transporter integral membrane Type 1 and ABC transporter family domains, respectively (Figure 2c). The c.373dupA variant introduced a premature stop codon at Position 158, truncating 1322 amino acids at the C terminus of the CFTR protein, whereas c.1342delA introduced a stop codon at Position 448, truncating 1032 amino acids at the C terminus (Figure 2c). Structural predictions using AlphaFold suggested that the p.Leu145Val variant in the ABC transporter integral membrane Type 1 domain might alter the orientation of tightly packed hydrophobic cores (Figure 2d). Additionally, the Lys968Asn substitution in the ABC transporter family domain was predicted to change hydrophobic interactions, inducing conformational shifts in the transmembrane domain (Figure 2d).

In ADGRG2, the c.959delCinsTT variant is predicted to introduce a premature stop codon at Position 323, resulting in the truncation of 694 amino acids at the C‐terminus. Similarly, the c.1402C > T variant introduces a stop codon at Position 468, leading to the loss of 549 amino acids (Figure 2c). The truncated region in the ADGRG2 protein includes the entire G‐protein–coupled receptor (GAIN‐B) domain (Figure 2c).

According to the 2015 ACMG Standards and Guidelines [9], these two novel nonsense variants, along with two frameshift variants in CFTR and ADGRG2, were classified as pathogenic. Additionally, the c.433C > G variant was classified as likely pathogenic, whereas c.2904A > C was designated as a variant of uncertain significance (Table S1).

### 3.4. RNA Splicing Analysis

To further investigate potential splicing variants not identified in functional analysis, minigene analysis showed that four variants at the intron–exon junctions of the CFTR gene, including c.579 + 4 T > C, c.1210 − 6 T > A, and c.3469 − 3C > A in iCAVD patients, and c.2908 + 4C > A in female partners, did not induce aberrant splicing (Figure 3). Based on the ACMG Standards and Guidelines [10], these four variants were classified as likely benign (Table S1).

Figure 3Splicing analysis. RT‐PCR is conducted using RNA extracted from transfected HKE293T cells. Subsequently, agarose gel electrophoresis and Sanger sequencing illustrate the impacts of variants on splicing. (a) CFTR c.579 + 4 T > C; (b) CFTR c.1210 − 6 T > A; (c) CFTR c.2908 + 4C > A; (d) CFTR c.3469 − 3C > A.  ^“^M^”^ = marker,  ^“^WT^”^ = wild type,  ^“^MUT^”^ = mutant.

**Figure:**
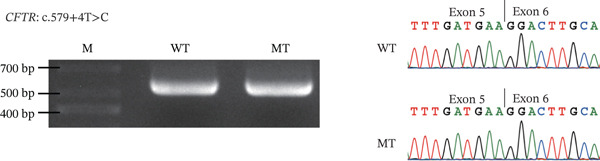
(a)

**Figure:**
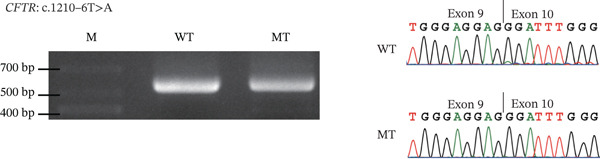
(b)

**Figure:**
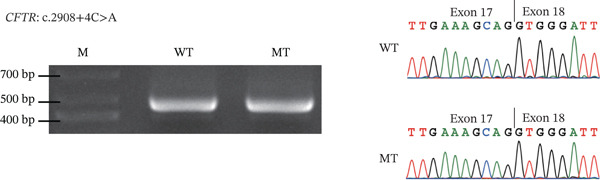
(c)

**Figure:**
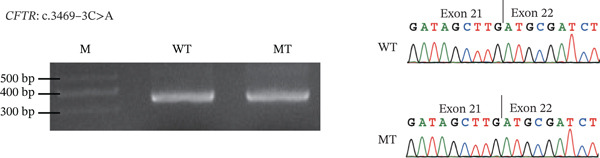
(d)

### 3.5. Association of Phenotypes and Genotypes in 199 iCAVD Patients

Among the 199 iCAVD patients, 122 out of 159 CBAVD patients (76.73%) and 27 out of 40 CUAVD patients (67.50%) were found to carry at least one CFTR or ADGRG2 variant. Furthermore, 64.15% (102/159) of CBAVD patients and 45.00% (18/40) of CUAVD patients carried at least two variants in CFTR or ADGRG2 (Figure 1b and Table S1). In total, 239 variants were detected across the 199 iCAVD patients, excluding 3 likely benign variants. Of these, 84.10% (201/239) of variants were identified in CBAVD patients, whereas 15.90% (38/239) were found in CUAVD patients (Figure 1b and Table S1). The average frequency of CFTR variants in CBAVD patients was 1.26 variants per patient (201/159), whereas CUAVD patients carried an average of 0.95 variants per patient (38/40).

To explore genotype–phenotype correlations, we stratified the entire cohort into three genetic subgroups based on CFTR and ADGRG2 variant profiles (Group I: no variants in either gene, Group II: single CFTR variant without ADGRG2 variants, and Group III: ≥ 2 CFTR variants or hemizygous ADGRG2 variant) and further subdivided these subgroups by phenotypic status ([CBAVD, n = 159] vs. [CUAVD, n = 40]) to account for phenotypic heterogeneity. In the CBAVD cohort, genotype‐defined groups differed in multiple phenotypic measures: Semen pH decreased stepwise from Group I to Group III (p < 0.0001), semen volume differed across groups (p = 0.008) with Group III lower than Group II (0.90 vs. 1.45 mL; p = 0.010), and semen fructose varied (p = 0.012) with Group III higher than Group II (1.05 vs. 0.63; p = 0.041). INHB also differed significantly among groups (p < 0.001), being higher in Group III than in Group I (p = 0.015) and Group II (p = 0.002). By contrast, age, FSH, LH, and alpha‐glucosidase were not significantly different (Table 2). In the CUAVD cohort, genotype–phenotype differences were limited to semen pH (p = 0.024), with Group III showing a lower pH than Group I (6.20 vs. 6.70; p = 0.031), whereas semen volume, fructose, alpha‐glucosidase, INHB, and other endocrine parameters did not differ (Table 2). Overall, genotype‐defined phenotypic variation was broader in CBAVD, but largely confined to semen pH in CUAVD.

**Table 2:** Comparison of clinical parameters among three groups of CBAVD and CUAVD.

Variables	Group I	Group II	Group III	Kruskal–Wallis	*p* values	*p*values(DSCF)
CBAVD (N = 159)	N = 38	N = 24	N = 97			
Age	30.00 (28.00, 34.00)	32.00 (30.00, 37.00)	31.00 (28.00, 34.00)	3.29	0.193	
FSH (IU/L)	4.40 (3.28, 5.32)	5.57 (3.67, 6.71)	4.82 (3.77, 6.07)	4.11	0.128	
LH (IU/L)	3.73 (2.87, 4.03)	3.88 (2.53, 4.86)	3.69 (2.86, 5.00)	0.67	0.715	
INHB (pg/mL) ^∗^	129.65 (116.00, 135.00)	124.02 (108.00, 125.00)	134.60 (135.00, 140.00)	16.58	≤ 0.001	I versus III: 0.015, II versus III: 0.002
Semen volume (mL) ^∗^	1.10 (0.70, 1.22)	1.45 (0.85, 1.72)	0.90 (0.70, 1.30)	9.55	0.008	II versus III: 0.010
PH ^∗^	6.70 (6.40, 7.00)	6.40 (6.20, 6.40)	6.20 (6.00, 6.40)	22.24	< 0.0001	I versus III < 0.0001, I versus II: 0.031
Fructose(umol/ejaculate) ^∗^	0.54 (0.30, 0.96)	0.63 (0.33, 0.63)	1.05 (0.44, 1.54)	8.80	0.012	I versus III: 0.055, II versus III: 0.041
Alpha‐glucosidase (mU/ejaculate)	2.11 (1.50,2.70)	2.35 (1.77, 2.38)	2.11 (1.69, 2.32)	3.36	0.187	
CUAVD (N = 40)	N = 13	N = 9	N = 18			
Age	30.00 (28.00, 33.00)	34.00 (30.00, 36.00)	30.50 (28.00, 33.00)	2.46	0.292	
FSH (IU/L)	4.40 (4.12, 5.97)	5.57 (5.57, 5.69)	4.82 (3.55, 5.70)	2.08	0.353	
LH (IU/L)	3.73 (3.73, 4.90)	3.88 (3.06, 3.88)	3.69 (2.90, 5.56)	0.32	0.854	
INHB (pg/mL)	129.65 (130, 155)	124.02 (124.00, 124.00)	134.60 (101.00, 169.00)	1.70	0.427	
Semen volume (mL)	1.40 (1.10, 2.29)	1.45 (0.90, 1.60)	0.90 (0.51, 1.50)	4.05	0.132	
PH ^∗^	6.70 (6.70, 7.20)	6.40 (6.40, 7.00)	6.20 (6.00, 6.70)	7.47	0.024	I versus III: 0.031
Fructose(umol/ejaculate)	0.54 (0.54, 0.54)	0.64 (0.63, 1.73)	1.05 (0.72, 1.05)	2.43	0.297	
Alpha‐glucosidase (mU/ejaculate)	2.11 (2.11, 2.11)	2.35 (1.32, 3.65)	2.11 (1.44, 2.11)	0.97	0.617	

*Note:* Normal range: FSH (IU/L), 1.27–19.26; LH (IU/L), 1.24–8.62; inhibin B (pg/mL), 18.22–311.27; semen volume (mL), 1.5–6.8; semen pH, 7.2–8.0; semen fructose (*μ*mol per ej): ≥ 13; semen alpha‐glucosidase (mU per ej): ≥ 20.

Abbreviations: DSCF, Dwass–Steel–Critchlow–Fligner; ej, ejaculation; FSH, follicle‐stimulating hormone; INHB, inhibin B; LH, luteinizing hormone; pH, pondus hydrogenii.

^∗^Means p value <0.05.

## 4. Discussion

In this study, we present the first comprehensive clinical and genetic investigation of iCAVD and their partners. We analyzed all exons of the CFTR and ADGRG2 genes in 199 iCAVD patients and performed CFTR genotyping in 148 female partners. Among the three common CFTR variants observed in Chinese iCAVD patients (5T, c.4056G > C, and c.1666A > G), 5T and c.4056G > C were both identified as pathogenic. We also discovered five novel variants and determined that four predicted splicing variants did not affect splicing function. Furthermore, CFTR genotyping in both iCAVD patients and their female partners revealed that 10.14% of couples shared a pathogenic or likely pathogenic variant. Lastly, significant differences were noted in the median and interquartile ranges for semen volume, semen pH, INHB, and fructose across different variant subgroups.

In our study, 149 patients (74.87%) carried one or more variants in CFTR or ADGRG2, with 76.73% of CBAVD patients and 67.50% of CUAVD patients having at least one variant (Table S1). These findings are consistent with our previous smaller‐sample results [4]. We identified four novel CFTR variants (p.Ile125Asnfs∗34, p.Leu145Val, p.Ile448∗, and p.Lys968Asn) and two novel ADGRG2 variants (p.Pro320Leufs∗4 and p.Gln468∗). In CFTR, the p.Ile125Asnfs∗34 and p.Ile448∗ variants were predicted to produce truncated proteins by removing some or all of the domains required for ATP binding and hydrolysis, which drives chloride ion transport across membranes. As a result, it might abolish chloride pumping function. In ADGRG2, the p.Pro320Leufs∗4 and p.Gln468∗ variants also resulted in truncated proteins that removed domains responsible for epididymal fluid exchange, suggesting a complete loss of function. Based on ACMG guidelines [10], these nonsense and frameshift variants were classified as pathogenic. For the CFTR missense variants, p.Lys968Asn and p.Leu145Val both are located within highly conserved regions of the protein. The p.Leu145Val variant was found in trans with the known pathogenic p.Ala613Thr variant, supporting its classification as likely pathogenic. In contrast, p.Lys968Asn was detected in a female partner and was designated as a variant of uncertain significance due to insufficient supporting evidence (Table S1).

In addition, we examined four potential splicing variants in CFTR (c.579 + 4 T > C, c.1210 − 6 T > A, c.2908 + 4C > A, and c.3469 − 3C > A) and found that none induced aberrant splicing (Figure 2). These findings revised our previous bioinformatic predictions for the c.579 + 4 T > C and c.1210 − 6 T > A variants [4], which were based on SpliceAI′s predictive algorithm and minigene splicing analysis. The results also reaffirmed that for noncanonical splice site variants, a SpliceAI *Δ*score of 0.2 or higher suggested a greater likelihood of splicing dysfunction [13, 14].

Based on data from 199 iCAVD patients, 5T and missense variants remained the predominant variant types causing iCAVD in the Chinese population, consistent with our previous findings but distinct from patterns observed in Caucasian populations [4]. The most frequent variant identified was 5T (34.42%), followed by c.4056G > C (p.Gln1352His) (7.04%) and c.1666A > G (p.Ile556Val) (4.02%). When comparing these with male controls in the gnomAD database, the frequencies of 5T and c.4056G > C were significantly higher in the patient cohort, whereas c.1666A > G did not differ significantly. Genetic analyses of iCAVD family members showed that 5T and c.4056G > C were in trans with other pathogenic variants in eight and four patients, respectively (Table S3). However, c.1666A > G occurred in cis with a pathogenic variant in a single individual. Consequently, c.1666A > G was classified as likely benign, whereas c.4056G > C was designated as pathogenic.

Recent Chinese iCAVD studies consistently show that the CFTR allelic architecture in Chinese iCAVD differs from that reported in Caucasian cohorts, with 5T repeatedly identified as the most frequent risk allele. In our previous Chinese CAVD cohort (n = 72), the three most common alleles were 5T (36.8%, 53/144), p.Ile556Val (6.9%, 10/144), and p.Gln1352His (3.5%, 5/144) [4], and multiple independent Chinese studies have similarly confirmed 5T as the predominant allele while reporting modest cohort‐ or region‐dependent variation in the “next‐tier” hotspots (commonly including p.Gln1352His and p.Ile556Val, and in some cohorts p.Gly970Asp or other missense variants) [15–18]. Recent larger cohorts and meta‐analyses further support this pattern, suggesting a typical Chinese iCAVD hotspot ranking of 5T, p.Gln1352His, p.Ile556Val, and p.Gly970Asp, whereas in Caucasian CBAVD the most frequent alleles and compound genotypes are classically centered on p.Phe508del, 5T, and p.Arg117His [1, 19]. Importantly, the Chinese iCAVD spectrum also differs from Chinese CF. Aggregated analyses of Chinese CF cases report ethnicity‐specific hotspots, with p.Gly970Asp, c.1766 + 5G > T, and p.Ile1023Arg among the most frequent variants, whereas p.Phe508del—the predominant CF‐causing mutation in Caucasian CF (followed by variants such as p.Gly542∗ and p.Gly551Asp)—is rare in reported Chinese CF cases [20–22]. These population and phenotype‐dependent differences likely reflect both ancestry‐specific variant distributions (founder effects and long‐term persistence of population‐enriched alleles) and genotype–phenotype correlation across the CFTR disease spectrum [23]. Classic CF typically results from two CF‐causing variants, whereas iCAVD is widely recognized as a CFTR‐related disorder (CFTR‐RD) that more often involves one severe plus one mild variant or two mild/variably penetrant variants (e.g., 5T and CFTR‐RD–causing alleles), preserving sufficient residual CFTR function to avoid severe multiorgan CF manifestations [2]. Clinically, these observations underscore the need for population‐tailored CFTR testing or whole exome sequencing in Chinese iCAVD—ensuring coverage of 5T and Chinese‐enriched alleles (e.g., p.Q1352H and p.Gly970Asp)—and caution against relying solely on Caucasian hotspot panels when counseling couples and assessing reproductive risk.

In our study, 17 distinct CFTR variants were identified among 148 female partners. Of these, six were classified as pathogenic or likely pathogenic, nine as variants of uncertain significance, and two as likely benign. Among the pathogenic/likely pathogenic variants, p.Gln1352His was the most frequent, followed by the 5T allele (Table S1), which is generally considered a variant of varying clinical consequence (VCC) rather than a classic CF‐causing variant. Female partners in iCAVD couples also mainly harbor three clinically relevant CFTR variant categories—CF‐causing (severe loss‐of‐function), CFTR‐RD–causing (typically milder alleles), and variants of VCC (e.g., 5T). These classes differ mechanistically: CF‐causing variants usually markedly reduce or abolish CFTR protein/channel activity, whereas CFTR‐RD/VCC alleles often retain partial residual function and are more likely to present as reproductive‐tract–limited phenotypes (e.g., iCAVD) rather than classic multiorgan CF. Accumulating evidence from Chinese cohorts indicates that CFTR variants can be detected in CUAVD regardless of renal anomalies [4, 24], and that a substantial proportion of iOA cases carry CFTR and/or ADGRG2 variants [25], supporting a phenotype‐driven recommendation that men with CBAVD, CUAVD, or severe oligozoospermia/obstructive azoospermia should be offered CFTR (and ADGRG2) testing irrespective of unilateral/bilateral presentation. In our expanded cohort with rigorous variant classification, 10.14% of female partners carried pathogenic/likely pathogenic CFTR variants, underscoring the clinical value of partner screening and suggesting that targeted hotspot panels may underestimate carrier rates. Accordingly, partner screening should be considered whenever a male carries a clinically relevant CFTR genotype, as reproductive risk follows autosomal‐recessive inheritance and depends on the couple′s combined genotype. Clinically, because CBAVD frequently necessitates sperm retrieval and intracytoplasmic sperm injection (ICSI), preimplantation genetic testing for monogenic disorders (PGT‐M) is the most direct option when the partner carries a CF‐causing variant and the predicted risk of classic CF is substantial, whereas natural conception with prenatal diagnosis may be reasonable for selected nonazoospermic CUAVD cases or lower‐penetrance CFTR‐RD scenarios, consistent with international guidance [26]. From a cost‐effectiveness perspective, a tiered strategy is preferable: Test the affected male first using assays suited to the Chinese spectrum (including 5T and population‐enriched alleles), perform reflex partner testing when indicated, and prioritize PGT‐M mainly for high‐risk genotypes when assisted reproductive technology is already required.

Building on the genotype–phenotype patterns observed in our cohort, CBAVD patients showed higher CFTR/ADGRG2 variant‐carrying rates and a greater average number of variants per individual than CUAVD patients, supporting the view that CBAVD and CUAVD may involve partially distinct etiologic mechanisms and that a subset of CUAVD cases could be attributable to additional genetic factors or other unknown causes [3]. Clinically, reduced semen volume (< 1.5 mL), an acidic semen pH (< 7.0), and seminal plasma biochemical markers below reference ranges (fructose < 13 *μ*mol/ejaculate and alpha‐glucosidase < 20 mU/ejaculate, per our institutional standards) have been proposed as a nonpathognomonic warning triad for obstructive phenotypes [3]. Consistent with this framework, when CBAVD patients were stratified by genotype (Group I: no variants in CFTR/ADGRG2, Group II: a single CFTR variant, and Group III: ≥ 2 CFTR variants or a hemizygous ADGRG2 variant), significant between‐group differences were observed for semen volume, semen pH, INHB, and fructose (Table 2). Notably, Group III exhibited the lowest semen pH, with a clear stepwise shift toward acidification compared with Groups I and II (Table 2), indicating that CBAVD patients with multivariant CFTR genotypes or hemizygous ADGRG2 variants tend to present with more pronounced abnormalities in semen acidity. In contrast, within CUAVD, genotype‐defined subgroup differences were comparatively limited: Semen pH remained the only parameter showing a significant difference across groups, whereas semen volume, fructose, alpha‐glucosidase, and endocrine indices (including INHB) were largely stable (Table 2). Through Group III showed a statistically higher mean INHB than the other groups, all three CBAVD groups remained within the normal reference range, and no significant intergroup differences were observed in CUAVD, with means likewise normal. Given that decreased INHB is primarily linked to impaired spermatogenesis rather than isolated obstruction, the overall normal INHB profile in CAVD is consistent with the expected obstructive pathophysiology. The clinical significance and potential mechanisms underlying this modest between‐group variation require validation in larger cohorts. Together, these findings suggest that genotype‐based subgrouping reveals broader phenotypic heterogeneity in CBAVD than in CUAVD, and that semen pH may be the most sensitive phenotypic indicator distinguishing genotypic subgroups, particularly in CBAVD (Table 2).

This study is limited by its single‐center design and its cross‐sectional nature. Additional prospective, multicenter studies with larger cohorts are needed to validate and extend our results. Furthermore, certain variants classified as having uncertain significance require in vivo and in vitro functional analyses to fully elucidate their pathogenic roles.

## 5. Conclusion

Our findings indicate that 74.87% of Chinese iCAVD patients carry CFTR or ADGRG2 variants, including six novel variants. We further demonstrate that c.4056G > C (p.Gln1352His) should be considered a pathogenic variant commonly observed in this population. Genotype–phenotype correlation analysis suggests that semen pH might serve as a predictor of patients harboring two or more variants, underscoring its role as a potential biological warning sign. Moreover, CFTR screening in our CAVD couples revealed a 10.14% risk of shared variant carriage, highlighting the need for genetic counseling to guide clinical management and inform both prenatal and postnatal care.

## Author Contributions

P.Y., H.C., and Q.S. conceived and designed the study. P.Y. and L.Z. performed genetic analyses and functional studies of the splicing variant. Z.L., J.Z., and J.L. diagnosed patients and interpreted clinical data. S.W., S.X., Y.L., and T.Y. collected clinical samples. X.J. conducted statistical analyses. P.Y., Z.L., and L.Z. drafted and revised the manuscript. The authors consider that the first three authors P.Y., Z.L., and L.Z. should be regarded as joint first authors.

## Funding

This work was supported by the National Natural Science Foundation of China (81801431), Natural Science Foundation Project of Xinjiang Uygur Autonomous Region (2023D01C161), Natural Science Foundation of Guangdong Province (2021A1515011379, 2024A1515013264), High‐level talent introduction supporting scientific research funds of Guangdong Provincial People′s Hospital (KY012021441), Sun Yat‐Sen Clinical Research (SYS‐5010‐202410), Guangzhou Basic and Applied Basic Research Foundation (2023A04J2081), Clinical Collaboration Project of Integrated Traditional Chinese and Western Medicine for Major and Complex Discases‐Threatened Abortion ([2023] No.250), Key Program of Joint Funds of the National Natural Science Foundation of China (U24A20781), and Guangdong Second Provincial General Hospital Talent Introduction Research Startup Fund (2025‐DW‐KZ‐031‐01).

## Disclosure

All authors reviewed and approved the final manuscript and are accountable for the integrity of the work.

## Conflicts of Interest

The authors declare no conflicts of interest.

## Supporting Information

Additional supporting information can be found online in the Supporting Information section.

## Supporting information


**Supporting Information 1** Table S1: The profile of CFTR and ADGRG2 gene variants in our study population (199 Chinese patients diagnosed with iCAVD and 148 female partners).

**Supporting Information 2** Table S2: RT‐PCR primers for four variants at the intron‐exon junctions of the CFTR gene.

**Supporting Information 3** Table S3: Pedigree verification of CFTR variants in 13 couples.

## Data Availability

The data that support the findings of this study are available on request from the corresponding author. The data are not publicly available due to privacy or ethical restrictions.
