# Supplementary material for: Spectrum and Classification of CFTR and ADGRG2 Variants in Chinese Patients With Isolated CAVD: A Large Cohort Study and Risk Assessment of CFTR Variant Carriage in Couples
Source: Hum Mutat. 2026 May 25;2026:5588277. doi: 10.1155/humu/5588277 (PMC13199994; doi:10.1155/humu/5588277)
Supplement: Supplementary file 1 — Supporting Information 1 Table S1: The profile of CFTR and ADGRG2 gene variants in our study population (199 Chinese patients diagnosed with iCAVD and 148 female partners). [file HUMU-2026-5588277-s001.pdf]

Sample No.	Age	Diagnosis	cDNA	Protein	Genotype	Exon	Intron	Chr	Position（h	REVEL v1.3	CADD v1.7	PolyPhen-2	Mutation ta	SpliceAI	AF gnomAD	dbSNP	HGMD	CFTR2 (updated on April 7, 2023)	ClinVar	ACMG	ACMG_criteria
AV001	31	CBAVD	c.1210_12T[5] (c.1210-7_1210-6del)		het		9	7	117188683	NA	NA	NA	Deleterious	NA	NA	rs1805177	CX931174	Varying clinical consequence	VCV000242535.51 Pathogenic(13); Likely pathogenic(1); Uncertain significance(1); Likely benign(1)	P	PVS1_Strength,PM2_Supporting,PM3
AV001	31	CBAVD	c.4056G>C	p.Gln1352His	het	25		7	117304834	0.923	23.4	Probably damaging	Deleterious	NA	0.000611	rs113857788	CM931158	NA	VCV000007237.37 Conflicting interpretations of pathogenicity Pathogenic(2); Uncertain significance(10); Benign(1); Likely benign(2)	P	PS3,PS4_Moderate,PM2_Supporting,PM3,PP3_Moderate
AV001Q	31		NA	NA				7													
AV002	26	CBAVD	WT	WT				7													
AV002Q	26		NA	NA				7													
AV003	26	CBAVD	c.1210_12T[5] (c.1210-7_1210-6del)		het		9	7	117188683	NA	NA	NA	Deleterious	NA	NA	rs1805177	CX931174	Varying clinical consequence	VCV000242535.51 Pathogenic(13); Likely pathogenic(1); Uncertain significance(1); Likely benign(1)	P	PVS1_Strength,PM2_Supporting,PM3
AV003	26	CBAVD	c.2909G>A	p.Gly970Asp	het	18		7	117246728	0.985	32	Probably damaging	Deleterious	0.32	0.0002	rs386134230	CM990363	CF-causing	VCV000035854.26(Pathogenic)	P	PVS1_Strength,PS3,PM2_Supporting,PM3,PP3_Strong
AV003Q	29		WT	WT				7													
AV004	29	CBAVD	c.1666A>G	p.Ile556Val	het	12		7	117227874	0.440	19.69	Benign	Deleterious	NA	0.00223	rs75789129	CM920985	NA	VCV000007196.45 (Pathogenic(1); Uncertain significance(1); Benign(4); Likely benign(6))	LB	PS3_Supporting, PM2_Supporting, BS1, BP1
AV004Q	29		NA	NA				7													
AV005	36	CBAVD	c.1210_12T[5] (c.1210-7_1210-6del)		het		9	7	117188683	NA	NA	NA	Deleterious	NA	NA	rs1805177	CX931174	Varying clinical consequence	VCV000242535.51 Pathogenic(13); Likely pathogenic(1); Uncertain significance(1); Likely benign(1)	P	PVS1_Strength,PM2_Supporting,PM3
AV005	36	CBAVD	c.2036G>A	p.Trp679*	het	14		7	117232257	NA	36	NA	Deleterious	NA	NA	rs397508333	CM993861	CF-causing	VCV000053429.3 (Pathogenic/Likely pathogenic)	P	PVS1,PM2_Supporting,PM3,PP3
AV005Q	36		NA	NA				7													
AV006	32	CBAVD	WT	WT				7													
AV006Q	32		NA	NA				7													
AV007	32	CBAVD	c.1210_12T[5] (c.1210-7_1210-6del)		het		9	7	117188683	NA	NA	NA	Deleterious	NA	NA	rs1805177	CX931174	Varying clinical consequence	VCV000242535.51 Pathogenic(13); Likely pathogenic(1); Uncertain significance(1); Likely benign(1)	P	PVS1_Strength,PM2_Supporting,PM3
AV007Q	32		NA	NA				7													
AV008	33	CBAVD	c.1210_12T[5] (c.1210-7_1210-6del)		het		9	7	117188683	NA	NA	NA	Deleterious	NA	NA	rs1805177	CX931174	Varying clinical consequence	VCV000242535.51 Pathogenic(13); Likely pathogenic(1); Uncertain significance(1); Likely benign(1)	P	PVS1_Strength,PM2_Supporting,PM3
AV008	33	CBAVD	c.1040G>A	p.Arg347His	het	8		7	117180324	0.885	32	Probably damaging	Deleterious	NA	0.00001123	rs77932196	CM920152	CF-causing	NA	P	PS3,PM2_Supporting,PM3,PP3_Moderate
AV008Q	29		NA	NA				7													
AV009	28	CBAVD	c.1210_12T[5] (c.1210-7_1210-6del)		het		9	7	117188683	NA	NA	NA	Deleterious	NA	NA	rs1805177	CX931174	Varying clinical consequence	VCV000242535.51 Pathogenic(13); Likely pathogenic(1); Uncertain significance(1); Likely benign(1)	P	PVS1_Strength,PM2_Supporting,PM3
AV009Q	26		WT	WT				7													
AV010	30	CBAVD	WT	WT				7													
AV010Q	31		NA	NA				7													
AV011	27	CUAVD	c.1210_12T[5] (c.1210-7_1210-6del)		het		9	7	117188683	NA	NA	NA	Deleterious	NA	NA	rs1805177	CX931174	Varying clinical consequence	VCV000242535.51 Pathogenic(13); Likely pathogenic(1); Uncertain significance(1); Likely benign(1)	P	PVS1_Strength,PM2_Supporting,PM3
AV011	27	CUAVD	c.869+5G>A		het		7	7	117176732	NA	22.1	NA	Deleterious	0.86	NA	rs533959068	CS075093	NA	VCV000552934.12 Pathogenic(2); Likely pathogenic(3); Uncertain significance(1)	P	PVS1_Strength,PM2_Supporting,PP3
AV011Q	24		NA	NA				7													
AV012	27	CUAVD	WT	WT				7													
AV012Q	27		NA	NA				7													
AV013	28	CUAVD	c.1210_12T[5] (c.1210-7_1210-6del)		homo		9	7	117188683	NA	NA	NA	Deleterious	NA	NA	rs1805177	CX931174	Varying clinical consequence	VCV000242535.51 Pathogenic(13); Likely pathogenic(1); Uncertain significance(1); Likely benign(1)	P	PVS1_Strength,PM2_Supporting,PM3
AV013Q	26		NA	NA				7													
AV014	28	CBAVD	WT	WT				7													
AV014Q	27		WT	WT				7													
AV015	35	CBAVD	c.1210_12T[5] (c.1210-7_1210-6del)		het		9	7	117188683	NA	NA	NA	Deleterious	NA	NA	rs1805177	CX931174	Varying clinical consequence	VCV000242535.51 Pathogenic(13); Likely pathogenic(1); Uncertain significance(1); Likely benign(1)	P	PVS1_Strength,PM2_Supporting,PM3
AV015	35	CBAVD	c.1666A>G	p.Ile556Val	het	12		7	117227874	0.440	19.69	Benign	Deleterious	NA	0.00223	rs75789129	CM920985	NA	VCV000007196.45 (Pathogenic(1); Uncertain significance(1); Benign(4); Likely benign(6))	LB	PS3_Supporting, PM2_Supporting, BS1, BP1
AV015Q	36		NA	NA				7													
AV016	32	CBAVD	WT	WT				7													
AV016	32	CBAVD	c.2452A>T	p.Lys818*	hemi	26		X	19017276	NA	38	NA	Deleterious	NA	NA	NA	CM192220	NA	NA	P	PVS1,PM2_Supporting,PP3_Moderate
AV016Q	32		WT	WT				7													
AV017	31	CBAVD	WT	WT				7													
AV017	31	CBAVD	c.3023G>A	p.Arg1008Gln	hemi	29		X	19009013	0.140	25.9	Probably damaging	Benign	NA	0.000029	rs777226422	CM192221	NA	NA	VUS	PM2_Supporting,PP3
AV017Q	34		NA	NA				7													
AV018	36	CBAVD	c.1210_12T[5] (c.1210-7_1210-6del)		homo		9	7	117188683	NA	NA	NA	Deleterious	NA	NA	rs1805177	CX931174	Varying clinical consequence	VCV000242535.51 Pathogenic(13); Likely pathogenic(1); Uncertain significance(1); Likely benign(1)	P	PVS1_Strength,PM2_Supporting,PM3
AV018Q	36		NA	NA				7													
AV019	31	CBAVD	c.1210_12T[5] (c.1210-7_1210-6del)		het		9	7	117188683	NA	NA	NA	Deleterious	NA	NA	rs1805177	CX931174	Varying clinical consequence	VCV000242535.51 Pathogenic(13); Likely pathogenic(1); Uncertain significance(1); Likely benign(1)	P	PVS1_Strength,PM2_Supporting,PM3
AV019	31	CBAVD	c.1766+5G>T		het		13	7	117230498	NA	20.7	NA	Deleterious	0.69	0.00006	rs121908796	CS951375	CF-causing	VCV000048685.25 (Pathogenic)	P	PVS1_Strength,PM2_Supporting,PP3
AV019Q	31		c.1666A>G	p.Ile556Val	het	12		7	117227874	0.440	19.69	Benign	Deleterious	NA	0.00223	rs75789129	CM920985	NA	VCV000007196.45 (Pathogenic(1); Uncertain significance(1); Benign(4); Likely benign(6))	LB	PS3_Supporting, PM2_Supporting, BS1, BP1
AV020	26	CBAVD	c.1210_12T[5] (c.1210-7_1210-6del)		homo		9	7	117188683	NA	NA	NA	Deleterious	NA	NA	rs1805177	CX931174	Varying clinical consequence	VCV000242535.51 Pathogenic(13); Likely pathogenic(1); Uncertain significance(1); Likely benign(1)	P	PVS1_Strength,PM2_Supporting,PM3
AV020Q	26		NA	NA				7													
AV021	30	CUAVD	WT	WT				7													
AV021Q	30		NA	NA				7													
AV022	37	CBAVD	c.482A>G	p.Tyr161Cys	het	4		7	117171161	0.969	27.6	Probably damaging	Deleterious	NA	NA	rs397508730	CM044864	NA	VCV000053967.2 (Uncertain significance)	LP	PM2_Supporting,PM5,PP3_Strong
AV022	37	CBAVD	c.4056G>C	p.Gln1352His	het	25		7	117304834	0.923	23.4	Probably damaging	Deleterious	NA	0.000611	rs113857788	CM931158	NA	VCV000007237.37 Conflicting interpretations of pathogenicity Pathogenic(2); Uncertain significance(10); Benign(1); Likely benign(2)	P	PS3,PS4_Moderate,PM2_Supporting,PM3,PP3_Moderate
AV022Q	36		NA	NA				7													
AV023	29	CUAVD	c.4056G>C	p.Gln1352His	homo	25		7	117304834	0.923	23.4	Probably damaging	Deleterious	NA	0.000611	rs113857788	CM931158	NA	VCV000007237.37 Conflicting interpretations of pathogenicity Pathogenic(2); Uncertain significance(10); Benign(1); Likely benign(2)	P	PS3,PS4_Moderate,PM2_Supporting,PM3,PP3_Moderate
AV023Q	27		NA	NA				7													
AV024	25	CUAVD	c.1210_12T[5] (c.1210-7_1210-6del)		homo		9	7	117188683	NA	NA	NA	Deleterious	NA	NA	rs1805177	CX931174	Varying clinical consequence	VCV000242535.51 Pathogenic(13); Likely pathogenic(1); Uncertain significance(1); Likely benign(1)	P	PVS1_Strength,PM2_Supporting,PM3
AV024Q	25		NA	NA				7													
AV025	33	CBAVD	c.1210_12T[5] (c.1210-7_1210-6del)		homo		9	7	117188683	NA	NA	NA	Deleterious	NA	NA	rs1805177	CX931174	Varying clinical consequence	VCV000242535.51 Pathogenic(13); Likely pathogenic(1); Uncertain significance(1); Likely benign(1)	P	PVS1_Strength,PM2_Supporting,PM3
AV025Q	33		NA	NA				7													
AV026	41	CUAVD	c.1210_12T[5] (c.1210-7_1210-6del)		het		9	7	117188683	NA	NA	NA	Deleterious	NA	NA	rs1805177	CX931174	Varying clinical consequence	VCV000242535.51 Pathogenic(13); Likely pathogenic(1); Uncertain significance(1); Likely benign(1)	P	PVS1_Strength,PM2_Supporting,PM3
AV026	41	CUAVD	c.1766+5G>T		het		13	7	117230498	NA	20.7	NA	Deleterious	0.69	0.00006	rs121908796	CS951375	CF-causing	VCV000048685.25 (Pathogenic)	P	PVS1_Strength,PM2_Supporting,PP3
AV026Q	41		NA	NA				7													
AV027	26	CUAVD	WT	WT				7													
AV027Q	23		NA	NA				7													
AV028	42	CBAVD	WT	WT				7													
AV028Q	31		c.358G>A	p.Ala120Thr	het	4		7	117171037	0.787	25.2	Probably damaging	Deleterious	NA	NA	rs201958172	CM940242	Varying clinical consequence	NA	P	PM2_Supporting,PP3
AV029	33	CUAVD	c.1210_12T[5] (c.1210-7_1210-6del)		het		9	7	117188683	NA	NA	NA	Deleterious	NA	NA	rs1805177	CX931174	Varying clinical consequence	VCV000242535.51 Pathogenic(13); Likely pathogenic(1); Uncertain significance(1); Likely benign(1)	P	PVS1_Strength,PM2_Supporting,PM3
AV029	33	CUAVD	c.-34C>T		het		5'-UTR	7	117120115	NA	15.48	NA	Deleterious	NA	0.000003988	rs756314710	CR118489	NA	VCV000552672.8 (Pathogenic(1); Uncertain significance(2))	LP	PS3,PM2_Supporting,PM3
AV029Q	34		WT	WT				7													
AV030	31	CUAVD	WT	WT				7													
AV030Q	30		NA	NA				7													
AV031	32	CUAVD	c.1657C>T	p.Arg553*	het	12		7	117227865	NA	39	NA	Deleterious	NA	0.00002241	rs74597325	CM900054	CF-causing	VCV000007122.125 (Pathogenic)	P	PVS1_Strength,PM2_Supporting,PP3
AV031	32	CUAVD	c.4056G>C	p.Gln1352His	het	25		7	117304834	0.923	23.4	Probably damaging	Deleterious	NA	0.000611	rs113857788	CM931158	NA	VCV000007237.37 Conflicting interpretations of pathogenicity Pathogenic(2); Uncertain significance(10); Benign(1); Likely benign(2)	P	PS3,PS4_Moderate,PM2_Supporting,PM3,PP3_Moderate
AV031Q	28		NA	NA				7													
AV032	24	CBAVD	WT	WT				7													
AV032Q	24		NA	NA				7													
AV033	35	CUAVD	c.1210_12T[5] (c.1210-7_1210-6del)		homo		9	7	117188683	NA	NA	NA	Deleterious	NA	NA	rs1805177	CX931174	Varying clinical consequence	VCV000242535.51 Pathogenic(13); Likely pathogenic(1); Uncertain significance(1); Likely benign(1)	P	PVS1_Strength,PM2_Supporting,PM3
AV033Q	34		NA	NA				7													
AV034	22	CBAVD	c.1210_12T[5] (c.1210-7_1210-6del)		het		9	7	117188683	NA	NA	NA	Deleterious	NA	NA	rs1805177	CX931174	Varying clinical consequence	VCV000242535.51 Pathogenic(13); Likely pathogenic(1); Uncertain significance(1); Likely benign(1)	P	PVS1_Strength,PM2_Supporting,PM3
AV034	22	CBAVD	c.926C>G	p.Ala309Gly	het	8		7	117180210	0.590	23.8	Benign	Deleterious	NA	0.00004779	rs397508818	CM980334	NA	VCV000054086.10 (Pathogenic(1); Likely pathogenic(1); Uncertain significance(4))	VUS	PM2_Supporting,PM3,PP3
AV034Q	22		NA	NA				7													
AV035	40	CBAVD	c.1210_12T[5] (c.1210-7_1210-6del)		het		9	7	117188683	NA	NA	NA	Deleterious	NA	NA	rs1805177	CX931174	Varying clinical consequence	VCV000242535.51 Pathogenic(13); Likely pathogenic(1); Uncertain significance(1); Likely benign(1)	P	PVS1_Strength,PM2_Supporting,PM3
AV035	40	CBAVD	c.-34C>T		het		5'-UTR	7	117120115	NA	15.48	NA	Deleterious	NA	0.000003988	rs756314710	CR118489	NA	VCV000552672.8 (Pathogenic(1); Uncertain significance(2))	LP	PS3,PM2_Supporting,PM3
AV035Q	40		NA	NA				7													
AV036	30	CBAVD	c.1210_12T[5] (c.1210-7_1210-6del)		het		9	7	117188683	NA	NA	NA	Deleterious	NA	NA	rs1805177	CX931174	Varying clinical consequence	VCV000242535.51 Pathogenic(13); Likely pathogenic(1); Uncertain significance(1); Likely benign(1)	P	PVS1_Strength,PM2_Supporting,PM3
AV036	30	CBAVD	c.1210-6T>A		het		9	7	117188689	NA	9.971	NA	Deleterious	0.09	0.00004060	rs763339686	NA	NA	VCV000810947.9 (Uncertain significance(1); Likely benign(1))	LB	PM2_Supporting,BS3,BP4
AV036Q	30		NA	NA				7													
AV037	32	CBAVD	WT	WT				7													
AV037Q	29		WT	WT				7													
AV038	27	CUAVD	WT	WT				7													
AV038Q	27		NA	NA				7													
AV039	29	CUAVD	c.1210_12T[5] (c.1210-7_1210-6del)		homo		9	7	117188683	NA	NA	NA	Deleterious	NA	NA	rs1805177	CX931174	Varying clinical consequence	VCV000242535.51 Pathogenic(13); Likely pathogenic(1); Uncertain significance(1); Likely benign(1)	P	PVS1_Strength,PM2_Supporting,PM3
AV039Q	29		NA	NA				7													
AV040	34	CBAVD	c.1210_12T[5] (c.1210-7_1210-6del)		het		9	7	117188683	NA	NA	NA	Deleterious	NA	NA	rs1805177	CX931174	Varying clinical consequence	VCV000242535.51 Pathogenic(13); Likely pathogenic(1); Uncertain significance(1); Likely benign(1)	P	PVS1_Strength,PM2_Supporting,PM3
AV040	34	CBAVD	c.1489A>G	p.Ile497Val	het	11		7	117199614	0.771	24.7	Possibly damaging	Deleterious	NA	NA	NA	CM024675	NA	NA	VUS	PM2_Supporting,PP3
AV040Q	27		WT	WT				7													
AV041	37	CBAVD	c.1210_12T[5] (c.1210-7_1210-6del)		homo		9	7	117188683	NA	NA	NA	Deleterious	NA	NA	rs1805177	CX931174	Varying clinical consequence	VCV000242535.51 Pathogenic(13); Likely pathogenic(1); Uncertain significance(1); Likely benign(1)	P	PVS1_Strength,PM2_Supporting,PM3
AV041Q	30		c.1210_12T[5] (c.1210-7_1210-6del)		het		9	7	117188683	NA	NA	NA	Deleterious	NA	NA	rs1805177	CX931174	Varying clinical consequence	VCV000242535.51 Pathogenic(13); Likely pathogenic(1); Uncertain significance(1); Likely benign(1)	P	PVS1_Strength,PM2_Supporting,PM3
AV042	34	CBAVD	c.-34C>T		het		5'-UTR	7	117120115	NA	15.48	NA	Deleterious	NA	0.000003988	rs756314710	CR118489	NA	VCV000552672.8 (Pathogenic(1); Uncertain significance(2))	LP	PS3,PM2_Supporting,PM3
AV042	34	CBAVD	c.558C>G	p.Asn186Lys	het	5		7	117174398	0.635	23.7	Probably damaging	Deleterious	NA	NA	rs397508753	CM151949	NA	VCV000558712.8 （Likely pathogenic(2); Uncertain significance(3)）	LP	PS1,PM2_Supporting,PP3
AV042Q	26		WT	WT				7													
AV043	40	CBAVD	c.1210_12T[5] (c.1210-7_1210-6del)		comhet		9	7	117188683	NA	NA	NA	Deleterious	NA	NA	rs1805177	CX931174	Varying clinical consequence	VCV000242535.51 Pathogenic(13); Likely pathogenic(1); Uncertain significance(1); Likely benign(1)	P	PVS1_Strength,PM2_Supporting,PM3
AV043	40	CBAVD	c.1394C>A	p.Thr465Asn	comhet	11		7	117199519	0.801	29.4	Probably damaging	Deleterious	NA	3.981E-06	rs758900656	CM063897	NA	VCV000495896.13 （Likely pathogenic(4); Uncertain significance(2)）	LP	PM2_Supporting,PM3,PM5,PP3_Moderate
AV043Q	42		c.4056G>C	p.Gln1352His	het	25		7	117304834	0.923	23.4	Probably damaging	Deleterious	NA	0.000611	rs113857788	CM931158	NA	VCV000007237.37 Conflicting interpretations of pathogenicity Pathogenic(2); Uncertain significance(10); Benign(1); Likely benign(2)	P	PS3,PS4_Moderate,PM2_Supporting,PM3,PP3_Moderate
AV044	28	CUAVD	WT	WT				7													

AV044Q	28		NA	NA				7													
AV045	42	CUAVD	WT	WT				7													
AV045Q	42		c.4056G>C	p.Gln1352His	het	25		7	117304834	0.923	23.4	Probably damaging	Deleterious	NA	0.000611	rs113857788	CM931158	NA	VCV000007237.37 Conflicting interpretations of pathogenicity Pathogenic(2); Uncertain significance(10); Benign(1); Likely benign(2)	P	PS3,PS4_Moderate,PM2_Supporting,PM3,PP3_Moderate
AV046	37	CBAVD	c.263T>G	p.Leu88*	het	3		7	117149186	NA	37	NA	Deleterious	NA	NA	rs397508412	CM920142	CF-causing	VCV000053534.12 （Pathogenic ）	P	PVS1_Strength,PM2_Supporting,PP3
AV046	37	CBAVD	c.4056G>C	p.Gln1352His	het	25		7	117304834	0.923	23.4	Probably damaging	Deleterious	NA	0.000611	rs113857788	CM931158	NA	VCV000007237.37 Conflicting interpretations of pathogenicity Pathogenic(2); Uncertain significance(10); Benign(1); Likely benign(2)	P	PS3,PS4_Moderate,PM2_Supporting,PM3,PP3_Moderate
AV046Q	35		WT	WT				7													
AV047	27	CUAVD	c.558C>G	p.Asn186Lys	het	5		7	117174398	0.635	23.7	Probably damaging	Deleterious	NA	NA	rs397508753	CM151949	NA	VCV000558712.8 （Likely pathogenic(2); Uncertain significance(3)）	LP	PS1,PM2_Supporting,PP3
AV047	27	CUAVD	c.4056G>C	p.Gln1352His	het	25		7	117304834	0.923	23.4	Probably damaging	Deleterious	NA	0.000611	rs113857788	CM931158	NA	VCV000007237.37 Conflicting interpretations of pathogenicity Pathogenic(2); Uncertain significance(10); Benign(1); Likely benign(2)	P	PS3,PS4_Moderate,PM2_Supporting,PM3,PP3_Moderate
AV047Q	27		NA	NA				7													
AV048	31	CBAVD	c.579+4T>C		het		5	7	117174423	NA	18.2	NA	Benign	0	NA	NA	CS192217	NA	VCV001749657.2 (Uncertain significance)	LB	PM2_Supporting,BS3,BP4
AV048	31	CBAVD	c.579+5G>A		het		5	7	117174424	NA	23.8	NA	Deleterious	0.48	NA	rs78440224	CS941440	CF-causing	VCV000054012.16 (Pathogenic)	P	PVS1_Strength,PM2_Supporting,PP3
AV048Q	29		WT	WT				7													
AV049	29	CBAVD	c.1210_12T[5] (c.1210-7_1210-6del)		het		9	7	117188683	NA	NA	NA	Deleterious	NA	NA	rs1805177	CX931174	Varying clinical consequence	VCV000242535.51 Pathogenic(13); Likely pathogenic(1); Uncertain significance(1); Likely benign(1)	P	PVS1_Strength,PM2_Supporting,PM3
AV049	29	CBAVD	c.869+3A>T		het		7	7	117176730	NA	17.32	NA	Deleterious	0.75	NA	rs1554380828	CS043635	NA	VCV000550999.5 (Pathogenic/Likely pathogenic)	P	PVS1_Strength,PM2_Supporting,PM3,PP3
AV049Q	30		c.1210_12T[5] (c.1210-7_1210-6del)		het		9	7	117188683	NA	NA	NA	Deleterious	NA	NA	rs1805177	CX931174	Varying clinical consequence	VCV000242535.51 Pathogenic(13); Likely pathogenic(1); Uncertain significance(1); Likely benign(1)	P	PVS1_Strength,PM2_Supporting,PM3
AV050	27	CBAVD	c.1210_12T[5] (c.1210-7_1210-6del)		het		9	7	117188683	NA	NA	NA	Deleterious	NA	NA	rs1805177	CX931174	Varying clinical consequence	VCV000242535.51 Pathogenic(13); Likely pathogenic(1); Uncertain significance(1); Likely benign(1)	P	PVS1_Strength,PM2_Supporting,PM3
AV050	27	CBAVD	c.4056G>C	p.Gln1352His	het	25		7	117304834	0.923	23.4	Probably damaging	Deleterious	NA	0.000611	rs113857788	CM931158	NA	VCV000007237.37 Conflicting interpretations of pathogenicity Pathogenic(2); Uncertain significance(10); Benign(1); Likely benign(2)	P	PS3,PS4_Moderate,PM2_Supporting,PM3,PP3_Moderate
AV050Q	26		WT	WT				7													
AV051	26	CUAVD	c.2684G>A	p.Ser895Asn	het	17		7	117243612	0.351	5.990	Benign	Polymorphis	NA	0.0003255	rs201864483	CM004880	NA	VCV000053543.23Uncertain significance(7); Benign(1); Likely benign(3)	VUS	PM2_Supporting,BP4
AV051Q	25		WT	WT				7													
AV052	39	CBAVD	c.1558G>A	p.Val520Ile	comhet	11		7	117199683	0.585	24.9	Benign	Deleterious	NA	0.0001522	rs77646904	CM941976	NA	VCV000035825.45 (Conflicting classifications of pathogenicity,Uncertain significance(13); Likely benign(1))	VUS	PM2_Supporting,PM5,PP3
AV052	39	CBAVD	c.2042A>T	p.Glu681Val	comhet	14		7	117232263	0.621	24.5	Possibly damaging	Deleterious	NA	0.0001355	rs201295415	CM1717571	NA	VCV000287205.19 (Uncertain significance(6); Likely benign(1))	VUS	PM2_Supporting,PP3
AV052Q	35		c.4056G>C	p.Gln1352His	het	25		7	117304834	0.923	23.4	Probably damaging	Deleterious	NA	0.000611	rs113857788	CM931158	NA	VCV000007237.37 Conflicting interpretations of pathogenicity Pathogenic(2); Uncertain significance(10); Benign(1); Likely benign(2)	P	PS3,PS4_Moderate,PM2_Supporting,PM3,PP3_Moderate
AV053	45	CBAVD	c.1231A>G	p.Lys411Glu	het	10		7	117188716	0.600	23.4	Possibly damaging	Deleterious	NA	0.0000283	rs748155731	CM1413656	CF-causing	VCV000502578.7 (Uncertain significance)	P	PM2_Supporting,PP3
AV053Q	36		WT	WT				7													
AV054	35	CBAVD	c.1210_12T[5] (c.1210-7_1210-6del)		het		9	7	117188683	NA	NA	NA	Deleterious	NA	NA	rs1805177	CX931174	Varying clinical consequence	VCV000242535.51 Pathogenic(13); Likely pathogenic(1); Uncertain significance(1); Likely benign(1)	P	PVS1_Strength,PM2_Supporting,PM3
AV054	35	CBAVD	c.865A>T	p.Arg289*	het	7		7	117176723	NA	37	NA	Deleterious	NA	NA	rs1057516619	CM1511150	CF-causing	VCV000370599.3 (Pathogenic/Likely pathogenic)	P	PVS1,PM2_Supporting,PP3
AV054Q	28		NA	NA				7	117188683		NA	NA	Deleterious	NA	NA	rs1805177	CX931174	Varying clinical consequence	VCV000242535.51 Pathogenic(13); Likely pathogenic(1); Uncertain significance(1); Likely benign(1)	P	PVS1_Strength,PM2_Supporting,PP3,PP4
AV055	25	CBAVD	c.1210_12T[5] (c.1210-7_1210-6del)		het		9	7	117188683	NA	NA	NA	Deleterious	NA	NA	rs1805177	CX931174	Varying clinical consequence	VCV000242535.51 Pathogenic(13); Likely pathogenic(1); Uncertain significance(1); Likely benign(1)	P	PVS1_Strength,PM2_Supporting,PM3
AV055	25	CBAVD	c.4056G>C	p.Gln1352His	het	25		7	117304834	0.923	23.4	Probably damaging	Deleterious	NA	0.000611	rs113857788	CM931158	NA	VCV000007237.37 Conflicting interpretations of pathogenicity Pathogenic(2); Uncertain significance(10); Benign(1); Likely benign(2)	P	PS3,PS4_Moderate,PM2_Supporting,PM3,PP3_Moderate
AV055Q	26		c.3085T>A	p.Leu1029Met	het	19		7	117250669	0.628	14.31	Probably damaging	Deleterious	NA	0.000003984	NA	NA	NA	VCV001705999.1 (Uncertain significance)	VUS	PM2_Supporting,PP3
AV056	32	CBAVD	c.1210_12T[5] (c.1210-7_1210-6del)		het		9	7	117188683	NA	NA	NA	Deleterious	NA	NA	rs1805177	CX931174	Varying clinical consequence	VCV000242535.51 Pathogenic(13); Likely pathogenic(1); Uncertain significance(1); Likely benign(1)	P	PVS1_Strength,PM2_Supporting,PM3
AV056	32	CBAVD	c.1407G>T	p.Met469Ile	het	11		7	117199532	0.821	26.1	Probably damaging	Deleterious	NA	NA	rs143218779	CM056306	NA	VCV000053249.2 (Uncertain significance)	VUS	PM2_Supporting,PP3_Moderate
AV056Q	32		NA	NA				7													
AV057	38	CBAVD	c.1210_12T[5] (c.1210-7_1210-6del)		homo		9	7	117188683	NA	NA	NA	Deleterious	NA	NA	rs1805177	CX931174	Varying clinical consequence	VCV000242535.51 Pathogenic(13); Likely pathogenic(1); Uncertain significance(1); Likely benign(1)	P	PVS1_Strength,PM2_Supporting,PM3
AV057	38	CBAVD	c.1666A>G	p.Ile556Val	het	12		7	117227874	0.440	19.69	Benign	Deleterious	NA	0.00223	rs75789129	CM920985	NA	VCV000007196.45 (Pathogenic(1); Uncertain significance(1); Benign(4); Likely benign(6))	LB	PS3_Supporting, PM2_Supporting, BS1, BP1
AV057Q	27		WT	WT				7													
AV058	41	CBAVD	c.1210_12T[6] (c.1210-6delT)		het		9	7	117188683	NA	NA	NA	Benign	0.02	NA	NA	NA	NA	VCV000495892.8 (Uncertain significance)	VUS	PM2_Supporting,BP4
AV058Q	40		c.1666A>G	p.Ile556Val	het	12		7	117227874	0.440	19.69	Benign	Deleterious	NA	0.00223	rs75789129	CM920985	NA	VCV000007196.45 (Pathogenic(1); Uncertain significance(1); Benign(4); Likely benign(6))	LB	PS3_Supporting, PM2_Supporting, BS1, BP1
AV059	32	CBAVD	c.1210_12T[5] (c.1210-7_1210-6del)		het		9	7	117188683	NA	NA	NA	Deleterious	NA	NA	rs1805177	CX931174	Varying clinical consequence	VCV000242535.51 Pathogenic(13); Likely pathogenic(1); Uncertain significance(1); Likely benign(1)	P	PVS1_Strength,PM2_Supporting,PM3
AV059Q	33		WT	WT				7													
AV060	28	CBAVD	WT	WT				7													
AV060Q	25		WT	WT				7													
AV061	47	CBAVD	c.1666A>G	p.Ile556Val	het	12		7	117227874	0.440	19.69	Benign	Deleterious	NA	0.00223	rs75789129	CM920985	NA	VCV000007196.45 (Pathogenic(1); Uncertain significance(1); Benign(4); Likely benign(6))	LB	PS3_Supporting, PM2_Supporting, BS1, BP1
AV061Q	38		WT	WT				7													
AV062	32	CBAVD	c.1210_12T[5] (c.1210-7_1210-6del)		homo		9	7	117188683	NA	NA	NA	Deleterious	NA	NA	rs1805177	CX931174	Varying clinical consequence	VCV000242535.51 Pathogenic(13); Likely pathogenic(1); Uncertain significance(1); Likely benign(1)	P	PVS1_Strength,PM2_Supporting,PM3
AV062	32	CBAVD	c.1666A>G	p.Ile556Val	het	12		7	117227874	0.440	19.69	Benign	Deleterious	NA	0.00223	rs75789129	CM920985	NA	VCV000007196.45 (Pathogenic(1); Uncertain significance(1); Benign(4); Likely benign(6))	LB	PS3_Supporting, PM2_Supporting, BS1, BP1
AV062Q	30		WT	WT				7													
AV063	32	CUAVD	c.1210_12T[5] (c.1210-7_1210-6del)		het		9	7	117188683	NA	NA	NA	Deleterious	NA	NA	rs1805177	CX931174	Varying clinical consequence	VCV000242535.51 Pathogenic(13); Likely pathogenic(1); Uncertain significance(1); Likely benign(1)	P	PVS1_Strength,PM2_Supporting,PM3
AV063Q	32		c.3205G>A	p.Gly1069Arg	het	20		7	117251700	0.603	23.5	Probably damaging	Deleterious	NA	0.00009556	rs200321110	CM003252	CF-causing	VCV000053684.40 (Pathogenic(2); Likely pathogenic(4); Uncertain significance(8))	P	PS3,PM2_Supporting,PM3,PP3
AV064	29	CBAVD	c.1210_12T[5] (c.1210-7_1210-6del)		het		9	7	117188683	NA	NA	NA	Deleterious	NA	NA	rs1805177	CX931174	Varying clinical consequence	VCV000242535.51 Pathogenic(13); Likely pathogenic(1); Uncertain significance(1); Likely benign(1)	P	PVS1_Strength,PM2_Supporting,PM3
AV064	29	CBAVD	c.1669T>A	p.Ser557Thr	het	12		7	117227877	0.696	22.4	Benign	Deleterious	NA	0.00001197	rs746642567	CM192216	NA	VCV001777639.2 (Uncertain significance)	VUS	PM2_Supporting,PM3,PP3
AV064Q	29		NA	NA				7													
AV065	31	CBAVD	c.1210_12T[5] (c.1210-7_1210-6del)		het		9	7	117188683	NA	NA	NA	Deleterious	NA	NA	rs1805177	CX931174	Varying clinical consequence	VCV000242535.51 Pathogenic(13); Likely pathogenic(1); Uncertain significance(1); Likely benign(1)	P	PVS1_Strength,PM2_Supporting,PM3
AV065Q	30		c.1210_12T[5] (c.1210-7_1210-6del)		het		9	7	117188683	NA	NA	NA	Deleterious	NA	NA	rs1805177	CX931174	Varying clinical consequence	VCV000242535.51 Pathogenic(13); Likely pathogenic(1); Uncertain significance(1); Likely benign(1)	P	PVS1_Strength,PM2_Supporting,PM3
AV066	33	CBAVD	c.1210_12T[5] (c.1210-7_1210-6del)		homo		9	7	117188683	NA	NA	NA	Deleterious	NA	NA	rs1805177	CX931174	Varying clinical consequence	VCV000242535.51 Pathogenic(13); Likely pathogenic(1); Uncertain significance(1); Likely benign(1)	P	PVS1_Strength,PM2_Supporting,PM3
AV066Q	32		NA	NA				7													
AV067	30	CBAVD	c.1210_12T[5] (c.1210-7_1210-6del)		het		9	7	117188683	NA	NA	NA	Deleterious	NA	NA	rs1805177	CX931174	Varying clinical consequence	VCV000242535.51 Pathogenic(13); Likely pathogenic(1); Uncertain significance(1); Likely benign(1)	P	PVS1_Strength,PM2_Supporting,PM3
AV067	30	CBAVD	c.4056G>C	p.Gln1352His	het	25		7	117304834	0.923	23.4	Probably damaging	Deleterious	NA	0.000611	rs113857788	CM931158	NA	VCV000007237.37 Conflicting interpretations of pathogenicity Pathogenic(2); Uncertain significance(10); Benign(1); Likely benign(2)	P	PS3,PS4_Moderate,PM2_Supporting,PM3,PP3_Moderate
AV067Q	30		WT	WT				7													
AV068	35	CBAVD	c.4056G>C	p.Gln1352His	het	25		7	117304834	0.923	23.4	Probably damaging	Deleterious	NA	0.000611	rs113857788	CM931158	NA	VCV000007237.37 Conflicting interpretations of pathogenicity Pathogenic(2); Uncertain significance(10); Benign(1); Likely benign(2)	P	PS3,PS4_Moderate,PM2_Supporting,PM3,PP3_Moderate
AV068Q	31		c.4056G>C	p.Gln1352His	het	25		7	117304834	0.923	23.4	Probably damaging	Deleterious	NA	0.000611	rs113857788	CM931158	NA	VCV000007237.37 Conflicting interpretations of pathogenicity Pathogenic(2); Uncertain significance(10); Benign(1); Likely benign(2)	P	PS3,PS4_Moderate,PM2_Supporting,PM3,PP3_Moderate
AV069	30	CUAVD	c.1210_12T[5] (c.1210-7_1210-6del)		het		9	7	117188683	NA	NA	NA	Deleterious	NA	NA	rs1805177	CX931174	Varying clinical consequence	VCV000242535.51 Pathogenic(13); Likely pathogenic(1); Uncertain significance(1); Likely benign(1)	P	PVS1_Strength,PM2_Supporting,PM3
AV069Q	30		NA	NA				7													
AV070	28	CBAVD	c.1210_12T[5] (c.1210-7_1210-6del)		comhet		9	7	117188683	NA	NA	NA	Deleterious	NA	NA	rs1805177	CX931174	Varying clinical consequence	VCV000242535.51 Pathogenic(13); Likely pathogenic(1); Uncertain significance(1); Likely benign(1)	P	PVS1_Strength,PM2_Supporting,PM3
AV070	28	CBAVD	c.865A>T	p.Arg289*	comhet	7		7	117176723	NA	37	NA	Deleterious	NA	NA	rs1057516619	CM1511150	CF-causing	VCV000370599.3 (Pathogenic/Likely pathogenic)	P	PVS1,PM2_Supporting,PP3
AV070Q	28		WT	WT				7													
AV071	37	CUAVD	c.1210_12T[5] (c.1210-7_1210-6del)		het		9	7	117188683	NA	NA	NA	Deleterious	NA	NA	rs1805177	CX931174	Varying clinical consequence	VCV000242535.51 Pathogenic(13); Likely pathogenic(1); Uncertain significance(1); Likely benign(1)	P	PVS1_Strength,PM2_Supporting,PM3
AV071Q	38		c.374T>C	p.Ile125Thr	het	4		7	117171053	0.668	22.2	Probably damaging	Deleterious	NA	0.0006978	rs141723617	CM983538	NA	VCV000053802.32 (Uncertain significance(6); Likely benign(4))	VUS	PM2_Supporting,PM3,PP3
AV072	35	CBAVD	c.1210_12T[5] (c.1210-7_1210-6del)		homo		9	7	117188683	NA	NA	NA	Deleterious	NA	NA	rs1805177	CX931174	Varying clinical consequence	VCV000242535.51 Pathogenic(13); Likely pathogenic(1); Uncertain significance(1); Likely benign(1)	P	PVS1_Strength,PM2_Supporting,PM3
AV072Q	35		WT	WT				7													
AV073	24	CBAVD	c.865A>T	p.Arg289*	het	7		7	117176723	NA	37	NA	Deleterious	NA	NA	rs1057516619	CM1511150	CF-causing	VCV000370599.3 (Pathogenic/Likely pathogenic)	P	PVS1,PM2_Supporting,PP3
AV073	24	CBAVD	c.4056G>C	p.Gln1352His	het	25		7	117304834	0.923	23.4	Probably damaging	Deleterious	NA	0.000611	rs113857788	CM931158	NA	VCV000007237.37 Conflicting interpretations of pathogenicity Pathogenic(2); Uncertain significance(10); Benign(1); Likely benign(2)	P	PS3,PS4_Moderate,PM2_Supporting,PM3,PP3_Moderate
AV073Q	26		c.2552G>A	p.Arg851Gln	het	15		7	117235045	0.799	29.3	Probably damaging	Deleterious	NA	0.00009907	rs397508395	CM970288	NA	VCV000411119.20 (Uncertain significance)	VUS	PM2_Supporting,PP3_Moderate
AV074	28	CBAVD	c.1210_12T[5] (c.1210-7_1210-6del)		het		9	7	117188683	NA	NA	NA	Deleterious	NA	NA	rs1805177	CX931174	Varying clinical consequence	VCV000242535.51 Pathogenic(13); Likely pathogenic(1); Uncertain significance(1); Likely benign(1)	P	PVS1_Strength,PM2_Supporting,PM3
AV074	28	CBAVD	c.2531C>T	p.Thr844Ile	het	15		7	117235024	0.680	26.5	Probably damaging	Deleterious	NA	NA	rs1792097477	NA	NA	VCV001422547.9 (Uncertain significance)	VUS	PM2_Supporting,PP3
AV074Q	26		WT	WT				7													
AV076	27	CBAVD	c.1210_12T[5] (c.1210-7_1210-6del)		comhet		9	7	117188683	NA	NA	NA	Deleterious	NA	NA	rs1805177	CX931174	Varying clinical consequence	VCV000242535.51 Pathogenic(13); Likely pathogenic(1); Uncertain significance(1); Likely benign(1)	P	PVS1_Strength,PM2_Supporting,PM3
AV076	27	CBAVD	c.4056G>C	p.Gln1352His	comhet	25		7	117304834	0.923	23.4	Probably damaging	Deleterious	NA	0.000611	rs113857788	CM931158	NA	VCV000007237.37 Conflicting interpretations of pathogenicity Pathogenic(2); Uncertain significance(10); Benign(1); Likely benign(2)	P	PS3,PS4_Moderate,PM2_Supporting,PM3,PP3_Moderate
AV076Q	27		WT	WT				7													
AV077	28	CBAVD	WT	WT				7													
AV077Q	25		WT	WT				7													
AV078	32	CUAVD	WT	WT				7													
AV078Q	28		WT	WT				7													
AV079	34	CBAVD	c.1210_12T[5] (c.1210-7_1210-6del)		homo		9	7	117188683	NA	NA	NA	Deleterious	NA	NA	rs1805177	CX931174	Varying clinical consequence	VCV000242535.51 Pathogenic(13); Likely pathogenic(1); Uncertain significance(1); Likely benign(1)	P	PVS1_Strength,PM2_Supporting,PM3
AV079Q	29		WT	WT				7													
AV080	26	CBAVD	WT	WT				7													
AV080Q	23		c.1666A>G	p.Ile556Val	het	12		7	117227874	0.440	19.69	Benign	Deleterious	NA	0.00223	rs75789129	CM920985	NA	VCV000007196.45 (Pathogenic(1); Uncertain significance(1); Benign(4); Likely benign(6))	LB	PS3_Supporting, PM2_Supporting, BS1, BP1
AV081	34	CBAVD	c.1210_12T[5] (c.1210-7_1210-6del)		het		9	7	117188683	NA	NA	NA	Deleterious	NA	NA	rs1805177	CX931174	Varying clinical consequence	VCV000242535.51 Pathogenic(13); Likely pathogenic(1); Uncertain significance(1); Likely benign(1)	P	PVS1_Strength,PM2_Supporting,PM3
AV081	34	CBAVD	c.558C>G	p.Asn186Lys	het	5		7	117174398	0.635	23.7	Probably damaging	Deleterious	NA	NA	rs397508753	CM151949	NA	VCV000558712.8 (Likely pathogenic(2); Uncertain significance(3))	LP	PS1,PM2_Supporting,PP3
AV081Q	30		WT	WT				7													
AV082	30	CBAVD	WT	WT				7													
AV082Q	27		c.1666A>G	p.Ile556Val	het	12		7	117227874	0.440	19.69	Benign	Deleterious	NA	0.00223	rs75789129	CM920985	NA	VCV000007196.45 (Pathogenic(1); Uncertain significance(1); Benign(4); Likely benign(6))	LB	PS3_Supporting, PM2_Supporting, BS1, BP1
AV083	28	CUAVD	WT	WT				7													
AV083Q	28		WT	WT				7													
AV084	26	CBAVD	c.3469-3C>A		het		21	7	117267573	NA	16.98	NA	Deleterious	0.19	0.00003994	NA	CS158229	NA	VCV001731705.5 (Uncertain significance)	LB	PM2_Supporting,BS3,BP4
AV084Q	27		c.1666A>G	p.Ile556Val	het	12		7	117227874	0.440	19.69	Benign	Deleterious	NA	0.00223	rs75789129	CM920985	NA	VCV000007196.45 (Pathogenic(1); Uncertain significance(1); Benign(4); Likely benign(6))	LB	PS3_Supporting, PM2_Supporting, BS1, BP1
AV085	30	CBAVD	c.1210_12T[5] (c.1210-7_1210-6del)		comhet		9	7	117188683	NA	NA	NA	Deleterious	NA	NA	rs1805177	CX931174	Varying clinical consequence	VCV000242535.51 Pathogenic(13); Likely pathogenic(1); Uncertain significance(1); Likely benign(1)	P	PVS1_Strength,PM2_Supporting,PM3
AV085	30	CBAVD	c.419C>G	p.Pro140Arg	comhet	4		7	117171098	0.833	25.8	Probably damaging	Deleterious	NA	NA	NA	NA	NA	NA	LP	PM2_Supporting,PM3,PM5_Supporting,PP3_Moderate
AV085Q	29		c.1666A>G	p.Ile556Val	comhet	12		7	117227874	0.440	19.69	Benign	Deleterious	NA	0.00223	rs75789129	CM920985	NA	VCV000007196.45 (Pathogenic(1); Uncertain significance(1); Benign(4); Likely benign(6))	LB	PS3_Supporting, PM2_Supporting, BS1, BP1
AV085Q	29		c.3205G>A	p.Gly1069Arg	comhet	20		7	117251700	0.603	23.5	Probably damaging	Deleterious	NA	0.00009556	rs200321110	CM003252	CF-causing	VCV000053684.40 (Pathogenic(2); Likely pathogenic(4); Uncertain significance(8))	P	PS3,PM2_Supporting,PM3,PP3
AV086	36	CBAVD	c.1210_12T[5] (c.1210-7_1210-6del)		het		9	7	117188683	NA	NA	NA	Deleterious	NA	NA	rs1805177	CX931174	Varying clinical consequence	VCV000242535.51 Pathogenic(13); Likely pathogenic(1); Uncertain significance(1); Likely benign(1)	P	PVS1_Strength,PM2_Supporting,PM3
AV086	36	CBAVD	c.2834C>T	p.Ser945Leu	het	17		7	117243762	0.949	32	Probably damaging	Deleterious	NA	0.00007072	rs397508442	CM930123	CF-causing	VCV000053575.35 (Pathogenic)	P	PS3,PM2_Supporting,PM3,PP3
AV086Q	33		WT	WT				7													
AV087	26	CBAVD	WT	WT				7													
AV087Q	26		NA	NA				7													

AV088	28	CBAVD	WT	WT				7													
AV088Q	28		NA	NA				7													
AV089	26	CBAVD	c.1210_12T[5] (c.1210-7_1210-6del)		homo		9	7	117188683	NA	NA	NA	Deleterious	NA	NA	rs1805177	CX931174	Varying clinical consequence	VCV000242535.51 Pathogenic(13); Likely pathogenic(1); Uncertain significance(1); Likely benign(1)	P	PVS1_Strength,PM2_Supporting,PM3
AV089Q	23		WT	WT				7													
AV090	31	CBAVD	c.1210_12T[5] (c.1210-7_1210-6del)		het		9	7	117188683	NA	NA	NA	Deleterious	NA	NA	rs1805177	CX931174	Varying clinical consequence	VCV000242535.51 Pathogenic(13); Likely pathogenic(1); Uncertain significance(1); Likely benign(1)	P	PVS1_Strength,PM2_Supporting,PM3
AV090Q	30		c.1666A>G	p.Ile556Val	het	12		7	117227874	0.440	19.69	Benign	Deleterious	NA	0.00223	rs75789129	CM920985	NA	VCV000007196.45 (Pathogenic(1); Uncertain significance(1); Benign(4); Likely benign(6))	LB	PS3_Supporting, PM2_Supporting, BS1, BP1
AV091	34	CBAVD	c.1210_12T[5] (c.1210-7_1210-6del)		het		9	7	117188683	NA	NA	NA	Deleterious	NA	NA	rs1805177	CX931174	Varying clinical consequence	VCV000242535.51 Pathogenic(13); Likely pathogenic(1); Uncertain significance(1); Likely benign(1)	P	PVS1_Strength,PM2_Supporting,PM3
AV091	34	CBAVD	c.296C>G	p.Pro99Arg	het	4		7	117170975	0.948	28.8	Probably damaging	Deleterious	NA	NA	NA	CM920980	CF-causing	VCV001704257.1 (Pathogenic)	P	PM2_Supporting,PP3
AV091Q	34		c.4056G>C	p.Gln1352His	het	25		7	117304834	0.923	23.4	Probably damaging	Deleterious	NA	0.000611	rs113857788	CM931158	NA	VCV000007237.37 Conflicting interpretations of pathogenicity Pathogenic(2); Uncertain significance(10); Benign(1); Likely benign(2)	P	PS3,PS4_Moderate,PM2_Supporting,PM3,PP3_Moderate
AV092	26	CBAVD	c.1210_12T[5] (c.1210-7_1210-6del)		homo		9	7	117188683	NA	NA	NA	Deleterious	NA	NA	rs1805177	CX931174	Varying clinical consequence	VCV000242535.51 Pathogenic(13); Likely pathogenic(1); Uncertain significance(1); Likely benign(1)	P	PVS1_Strength,PM2_Supporting,PM3
AV092	26	CBAVD	c.374T>C	p.Ile125Thr	het	4		7	117171053	0.668	22.2	Probably damaging	Deleterious	NA	0.0006978	rs141723617	CM983538	NA	VCV000053802.32 (Uncertain significance(6); Likely benign(4))	VUS	PM2_Supporting,PM3,PP3
AV092Q	24		WT	WT				7													
AV093	29	CBAVD	c.-34C>T		het		5'-UTR	7	117120115	NA	14.38	NA	Deleterious	NA	0.000003988	rs756314710	CR118489	NA	VCV000552672.8 (Pathogenic(1); Uncertain significance(2))	LP	PS3,PM2_Supporting,PM3
AV093	29	CBAVD	c.4056G>C	p.Gln1352His	het	25		7	117304834	0.923	23.4	Probably damaging	Deleterious	NA	0.000611	rs113857788	CM931158	NA	VCV000007237.37 Conflicting interpretations of pathogenicity Pathogenic(2); Uncertain significance(10); Benign(1); Likely benign(2)	P	PS3,PS4_Moderate,PM2_Supporting,PM3,PP3_Moderate
AV093Q	26		WT	WT				7													
AV094	31	CUAVD	c.1210_12T[5] (c.1210-7_1210-6del)		het		9	7	117188683	NA	NA	NA	Deleterious	NA	NA	rs1805177	CX931174	Varying clinical consequence	VCV000242535.51 Pathogenic(13); Likely pathogenic(1); Uncertain significance(1); Likely benign(1)	P	PVS1_Strength,PM2_Supporting,PM3
AV094	31	CUAVD	c.1766+5G>T		het		13	7	117230498	NA	20.7	NA	Deleterious	0.69	0.00006	rs121908796	CS951375	CF-causing	VCV000048685.25 (Pathogenic)	P	PVS1_Strength,PM2_Supporting,PP3
AV094Q	29		WT	WT				7													
AV095	35	CBAVD	c.1210_12T[5] (c.1210-7_1210-6del)		het		9	7	117188683	NA	NA	NA	Deleterious	NA	NA	rs1805177	CX931174	Varying clinical consequence	VCV000242535.51 Pathogenic(13); Likely pathogenic(1); Uncertain significance(1); Likely benign(1)	P	PVS1_Strength,PM2_Supporting,PM3
AV095	35	CBAVD	c.1657C>T	p.Arg553*	het	12		7	117227865	NA	39	NA	Deleterious	NA	0.00002241	rs74597325	CM900054	CF-causing	VCV000007122.125 (Pathogenic)	P	PVS1_Strength,PM2_Supporting,PP3
AV095Q	30		c.1210_12T[5] (c.1210-7_1210-6del)		het		9	7	117188683	NA	NA	NA	Deleterious	NA	NA	rs1805177	CX931174	Varying clinical consequence	VCV000242535.51 Pathogenic(13); Likely pathogenic(1); Uncertain significance(1); Likely benign(1)	P	PVS1_Strength,PM2_Supporting,PM3
AV096	26	CBAVD	c.1453A>T	p.Ser485Cys	het	11		7	117199578	0.912	25.7	Probably damaging	Deleterious	NA	0.00005305	rs138427145	CM993857	NA	VCV000053258.6 (Uncertain significance)	VUS	PM2_Supporting,PP3_Moderate
AV096	26	CBAVD	c.2812G>T	p.Val938Leu	het	17		7	117243740	0.640	24.7	Probably damaging	Deleterious	NA	0.00002387	rs749784731	NA	NA	VCV000928496.10 (Uncertain significance)	VUS	PM2_Supporting,PP3
AV096Q	25		WT	WT				7													
AV097	33	CBAVD	WT	WT				7													
AV097Q	33		NA	NA				7													
AV098	30	CUAVD	c.1210_12T[5] (c.1210-7_1210-6del)		homo		9	7	117188683	NA	NA	NA	Deleterious	NA	NA	rs1805177	CX931174	Varying clinical consequence	VCV000242535.51 Pathogenic(13); Likely pathogenic(1); Uncertain significance(1); Likely benign(1)	P	PVS1_Strength,PM2_Supporting,PM3
AV098Q	30		NA	NA				7													
AV099	36	CUAVD	c.2042A>T	p.Glu681Val	het	14		7	117232263	0.621	24.5	Possibly damaging	Deleterious	NA	0.0001355	rs201295415	NA	NA	VCV000287205.18 (Uncertain significance(6); Likely benign(1))	VUS	PM2_Supporting,PP3
AV099Q	36		WT	WT				7													
AV100	33	CUAVD	WT	WT				7													
AV100Q	33		WT	WT				7													
AV101	32	CBAVD	c.1210_12T[5] (c.1210-7_1210-6del)		het		9	7	117188683	NA	NA	NA	Deleterious	NA	NA	rs1805177	CX931174	Varying clinical consequence	VCV000242535.51 Pathogenic(13); Likely pathogenic(1); Uncertain significance(1); Likely benign(1)	P	PVS1_Strength,PM2_Supporting,PM3
AV101	32	CBAVD	c.2083_2084insG	p.Glu695GlyfsTer35	het	14		7	117232304_	NA	NA	NA	Deleterious	NA	NA	rs397508339	CI003302	NA	VCV000053437.9 (Pathogenic)	P	PVS1_Strength,PM2_Supporting,PP3
AV101	32	CBAVD	c.2684G>A	p.Ser895Asn	het	17		7	117243612	0.351	5.990	Benign	Polymorphis	NA	0.0003255	rs201864483	CM004880	NA	VCV000053543.23 Uncertain significance(7); Benign(1); Likely benign(3)	VUS	PM2_Supporting,BP4
AV101Q	33		WT	WT				7													
AV102	31	CBAVD	c.1210_12T[5] (c.1210-7_1210-6del)		het		9	7	117188683	NA	NA	NA	Deleterious	NA	NA	rs1805177	CX931174	Varying clinical consequence	VCV000242535.51 Pathogenic(13); Likely pathogenic(1); Uncertain significance(1); Likely benign(1)	P	PVS1_Strength,PM2_Supporting,PM3
AV102	31	CBAVD	c.1402C>T	p.Gln468*	hemi	18		X	19027764	NA	35	NA	Deleterious	NA	NA	rs2060312223	NA	NA	NA	P	PVS1,PM2_Supporting,PP3
AV102Q	31		WT	WT				7													
AV103	35	CBAVD	WT	WT				7													
AV103Q	35		WT	WT				7													
AV104	25	CBAVD	c.1210_12T[5] (c.1210-7_1210-6del)		homo		9	7	117188683	NA	NA	NA	Deleterious	NA	NA	rs1805177	CX931174	Varying clinical consequence	VCV000242535.51 Pathogenic(13); Likely pathogenic(1); Uncertain significance(1); Likely benign(1)	P	PVS1_Strength,PM2_Supporting,PM3
AV104Q	29		WT	WT				7													
AV105	32	CBAVD	c.2909G>A	p.Gly970Asp	het	18		7	117246728	0.985	32	Probably damaging	Deleterious	0.32	0.0002	rs386134230	CM990363	CF-causing	VCV000035854.26 (Pathogenic)	P	PVS1_Strength,PS3,PM2_Supporting,PM3,PP3
AV105	32	CBAVD	c.4056G>C	p.Gln1352His	het	25		7	117304834	0.923	23.4	Probably damaging	Deleterious	NA	0.000611	rs113857788	CM931158	NA	VCV000007237.37 Conflicting interpretations of pathogenicity Pathogenic(2); Uncertain significance(10); Benign(1); Likely benign(2)	P	PS3,PS4_Moderate,PM2_Supporting,PM3,PP3_Moderate
AV105Q	33		WT	WT				7													
AV106	31	CBAVD	c.1210_12T[5] (c.1210-7_1210-6del)		homo		9	7	117188683	NA	NA	NA	Deleterious	NA	NA	rs1805177	CX931174	Varying clinical consequence	VCV000242535.51 Pathogenic(13); Likely pathogenic(1); Uncertain significance(1); Likely benign(1)	P	PVS1_Strength,PM2_Supporting,PM3
AV106Q	31		WT	WT				7													
AV107	39	CBAVD	c.1210_12T[5] (c.1210-7_1210-6del)		het		9	7	117188683	NA	NA	NA	Deleterious	NA	NA	rs1805177	CX931174	Varying clinical consequence	VCV000242535.51 Pathogenic(13); Likely pathogenic(1); Uncertain significance(1); Likely benign(1)	P	PVS1_Strength,PM2_Supporting,PM3
AV107	39	CBAVD	c.1766+5G>T		het		13	7	117230498	NA	20.7	NA	Deleterious	0.69	0.00006	rs121908796	CS951375	CF-causing	VCV000048685.25 (Pathogenic)	P	PVS1_Strength,PM2_Supporting,PP3
AV107Q	38		NA	NA				7													
AV108	28	CBAVD	c.1210_12T[5] (c.1210-7_1210-6del)		het		9	7	117188683	NA	NA	NA	Deleterious	NA	NA	rs1805177	CX931174	Varying clinical consequence	VCV000242535.51 Pathogenic(13); Likely pathogenic(1); Uncertain significance(1); Likely benign(1)	P	PVS1_Strength,PM2_Supporting,PM3
AV108	28	CBAVD	c.4056G>C	p.Gln1352His	het	25		7	117304834	0.923	23.4	Probably damaging	Deleterious	NA	0.000611	rs113857788	CM931158	NA	VCV000007237.37 Conflicting interpretations of pathogenicity Pathogenic(2); Uncertain significance(10); Benign(1); Likely benign(2)	P	PS3,PS4_Moderate,PM2_Supporting,PM3,PP3_Moderate
AV108Q	26		WT	WT				7													
AV109	37	CBAVD	c.1666A>G	p.Ile556Val	het	12		7	117227874	0.440	19.69	Benign	Deleterious	NA	0.00223	rs75789129	CM920985	NA	VCV000007196.45 (Pathogenic(1); Uncertain significance(1); Benign(4); Likely benign(6))	LB	PS3_Supporting, PM2_Supporting, BS1, BP1
AV109Q	29		c.1666A>G	p.Ile556Val	het	12		7	117227874	0.440	19.69	Benign	Deleterious	NA	0.00223	rs75789129	CM920985	NA	VCV000007196.45 (Pathogenic(1); Uncertain significance(1); Benign(4); Likely benign(6))	LB	PS3_Supporting, PM2_Supporting, BS1, BP1
AV110	40	CBAVD	c.1210_12T[5] (c.1210-7_1210-6del)		homo		9	7	117188683	NA	NA	NA	Deleterious	NA	NA	rs1805177	CX931174	Varying clinical consequence	VCV000242535.51 Pathogenic(13); Likely pathogenic(1); Uncertain significance(1); Likely benign(1)	P	PVS1_Strength,PM2_Supporting,PM3
AV110Q	30		c.1210_12T[5] (c.1210-7_1210-6del)		het		9	7	117188683	NA	NA	NA	Deleterious	NA	NA	rs1805177	CX931174	Varying clinical consequence	VCV000242535.51 Pathogenic(13); Likely pathogenic(1); Uncertain significance(1); Likely benign(1)	P	PVS1_Strength,PM2_Supporting,PM3
AV111	29	CBAVD	WT	WT				7													
AV111Q	29		WT	WT				7													
AV112	28	CUAVD	WT	WT				7													
AV112Q	28		WT	WT				7													
AV113	36	CBAVD	WT	WT				7													
AV113Q	29		c.913T>A	p.Phe305Ile	het	8		7	117180197	0.483	19.88	Benign	Deleterious	NA	NA	NA	CM067754	NA	NA	VUS	PM2_Supporting
AV113Q	29		c.1666A>G	p.Ile556Val	het	12		7	117227874	0.440	19.69	Benign	Deleterious	NA	0.00223	rs75789129	CM920985	NA	VCV000007196.45 (Pathogenic(1); Uncertain significance(1); Benign(4); Likely benign(6))	LB	PS3_Supporting, PM2_Supporting, BS1, BP1
AV114	42	CBAVD	WT	WT				7													
AV114Q	42		WT	WT				7													
AV115	31	CBAVD	c.1210_12T[5] (c.1210-7_1210-6del)		het		9	7	117188683	NA	NA	NA	Deleterious	NA	NA	rs1805177	CX931174	Varying clinical consequence	VCV000242535.51 Pathogenic(13); Likely pathogenic(1); Uncertain significance(1); Likely benign(1)	P	PVS1_Strength,PM2_Supporting,PM3
AV115	31	CBAVD	c.2929T>C	p.Ser977Pro	het	18		7	117246748	0.867	25.4	Possibly damaging	Deleterious	NA	0.000003982	rs137975784	CM960285	NA	VCV000053599.2 (Likely pathogenic)	VUS	PM2_Supporting,PM5,PP3_Moderate
AV115Q	29		WT	WT				7													
AV116	27	CBAVD	c.1210_12T[5] (c.1210-7_1210-6del)		homo		9	7	117188683	NA	NA	NA	Deleterious	NA	NA	rs1805177	CX931174	Varying clinical consequence	VCV000242535.51 Pathogenic(13); Likely pathogenic(1); Uncertain significance(1); Likely benign(1)	P	PVS1_Strength,PM2_Supporting,PM3
AV116Q	25		WT	WT				7													
AV117	35	CBAVD	c.4056G>C	p.Gln1352His	het	25		7	117304834	0.923	23.4	Probably damaging	Deleterious	NA	0.000611	rs113857788	CM931158	NA	VCV000007237.37 Conflicting interpretations of pathogenicity Pathogenic(2); Uncertain significance(10); Benign(1); Likely benign(2)	P	PS3,PS4_Moderate,PM2_Supporting,PM3,PP3_Moderate
AV117Q	35		c.1666A>G	p.Ile556Val	het	12		7	117227874	0.440	19.69	Benign	Deleterious	NA	0.00223	rs75789129	CM920985	NA	VCV000007196.45 (Pathogenic(1); Uncertain significance(1); Benign(4); Likely benign(6))	LB	PS3_Supporting, PM2_Supporting, BS1, BP1
AV117Q	35		c.2684G>A	p.Ser895Asn	het	17		7	117243612	0.351	5.990	Benign	Polymorphis	NA	0.0003255	rs201864483	CM004880	NA	VCV000053543.23 Uncertain significance(7); Benign(1); Likely benign(3)	VUS	PM2_Supporting,BP4
AV118	38	CUAVD	c.1210_12T[5] (c.1210-7_1210-6del)		het		9	7	117188683	NA	NA	NA	Deleterious	NA	NA	rs1805177	CX931174	Varying clinical consequence	VCV000242535.51 Pathogenic(13); Likely pathogenic(1); Uncertain significance(1); Likely benign(1)	P	PVS1_Strength,PM2_Supporting,PM3
AV118Q	36		WT	WT				7													
AV119	24	CBAVD	WT	WT				7													
AV119Q	25		WT	WT				7													
AV120	22	CBAVD	c.1210_12T[5] (c.1210-7_1210-6del)		het		9	7	117188683	NA	NA	NA	Deleterious	NA	NA	rs1805177	CX931174	Varying clinical consequence	VCV000242535.51 Pathogenic(13); Likely pathogenic(1); Uncertain significance(1); Likely benign(1)	P	PVS1_Strength,PM2_Supporting,PM3
AV120	22	CBAVD	c.1943delA	p.Asp648Valfs*15	het	14		7	117232164	NA	NA	NA	Deleterious	NA	NA	rs1481564133	CD160260	CF-causing	VCV000634829.1 （Pathogenic ）	P	PVS1_Strength,PM2_Supporting,PP3
AV120Q	22		NA	NA				7													
AV121	33	CBAVD	c.1210_12T[5] (c.1210-7_1210-6del)		het		9	7	117188683	NA	NA	NA	Deleterious	NA	NA	rs1805177	CX931174	Varying clinical consequence	VCV000242535.51 Pathogenic(13); Likely pathogenic(1); Uncertain significance(1); Likely benign(1)	P	PVS1_Strength,PM2_Supporting,PM3
AV121	33	CBAVD	c.2812G>T	p.Val938Leu	het	17		7	117243740	0.640	24.7	Probably damaging	Deleterious	NA	0.00002387	rs749784731	NA	NA	VCV000928496.10 (Uncertain significance)	VUS	PM2_Supporting,PP3
AV121	33	CBAVD	c.2907A>C	p.Ala969=	het	17		7	117243835	NA	18.43	NA	Deleterious	0.44	0.00002389	rs377502207	CS165915	CF-causing	VCV000549917.10 (Likely pathogenic(1); Uncertain significance(3); Likely benign(1))	P	PVS1_Strength,PM2_Supporting,PP3
AV121Q	26		WT	WT				7													
AV122	29	CBAVD	c.1210_12T[5] (c.1210-7_1210-6del)		het		9	7	117188683	NA	NA	NA	Deleterious	NA	NA	rs1805177	CX931174	Varying clinical consequence	VCV000242535.51 Pathogenic(13); Likely pathogenic(1); Uncertain significance(1); Likely benign(1)	P	PVS1_Strength,PM2_Supporting,PM3
AV122	29	CBAVD	c.4056G>C	p.Gln1352His	het	25		7	117304834	0.923	23.4	Probably damaging	Deleterious	NA	0.000611	rs113857788	CM931158	NA	VCV000007237.37 Conflicting interpretations of pathogenicity Pathogenic(2); Uncertain significance(10); Benign(1); Likely benign(2)	P	PS3,PS4_Moderate,PM2_Supporting,PM3,PP3_Moderate
AV122Q	25		WT	WT				7													
AV123	29	CBAVD	c.1936G>T	p.Gly646*	het	14		7	117232157	NA	42	NA	Deleterious	NA	NA	rs1562907873	NA	CF-causing	VCV000618908.2 （Pathogenic ）	P	PM2_Supporting,PP3
AV123	29	CBAVD	c.3469-3C>A		het		21	7	117267573	NA	16.98	NA	Deleterious	0.19	0.00003994	NA	CS158229	NA	VCV001731705.5 (Uncertain significance)	LB	PM2_Supporting,PP3,BS3,BP4
AV123Q	29		WT	WT				7													
AV124	29	CBAVD	c.1210_12T[5] (c.1210-7_1210-6del)		homo		9	7	117188683	NA	NA	NA	Deleterious	NA	NA	rs1805177	CX931174	Varying clinical consequence	VCV000242535.51 Pathogenic(13); Likely pathogenic(1); Uncertain significance(1); Likely benign(1)	P	PVS1_Strength,PM2_Supporting,PM3
AV124Q	24		WT	WT				7													
AV125	31	CBAVD	c.1666A>G	p.Ile556Val	homo	12		7	117227874	0.440	19.69	Benign	Deleterious	NA	0.00223	rs75789129	CM920985	NA	VCV000007196.45 (Pathogenic(1); Uncertain significance(1); Benign(4); Likely benign(6))	LB	PS3_Supporting, PM2_Supporting, BS1, BP1
AV125Q	31		WT	WT				7													
AV126	31	CBAVD	c.1210_12T[5] (c.1210-7_1210-6del)		homo		9	7	117188683	NA	NA	NA	Deleterious	NA	NA	rs1805177	CX931174	Varying clinical consequence	VCV000242535.51 Pathogenic(13); Likely pathogenic(1); Uncertain significance(1); Likely benign(1)	P	PVS1_Strength,PM2_Supporting,PM3
AV126	31	CBAVD	c.1558G>A	p.Val520Ile	het	11		7	117199683	0.585	22.0	Benign	Deleterious	NA	0.0001522	rs77646904	CM941976	NA	VCV000035825.45 (Conflicting classifications of pathogenicity,Uncertain significance(13); Likely benign(1))	VUS	PM2_Supporting,PM5,PP3
AV126Q	31		WT	WT				7													
AV127	34	CBAVD	WT	WT				7													
AV127Q	33		WT	WT				7													
AV128	27	CBAVD	c.1210_12T[5] (c.1210-7_1210-6del)		het		9	7	117188683	NA	NA	NA	Deleterious	NA	NA	rs1805177	CX931174	Varying clinical consequence	VCV000242535.51 Pathogenic(13); Likely pathogenic(1); Uncertain significance(1); Likely benign(1)	P	PVS1_Strength,PM2_Supporting,PM3
AV128	27	CBAVD	c.233delT	p.Phe78Serfs*12	het	3		7	117149156	NA	NA	NA	Deleterious	NA	NA	rs397508360	CD983595	NA	VCV000053476.2 （Pathogenic ）	P	PVS1_Strength,PM2_Supporting,PP3
AV128Q	32		WT	WT				7													
AV129	39	CBAVD	c.3265T>C	p.Trp1089Arg	het	20		7	117251760	0.923	26.8	Probably damaging	Deleterious	NA	NA	NA	CM950254	NA	NA	LP	PS3,PM2_Supporting,PP3_Moderate
AV129	39	CBAVD	c.4056G>C	p.Gln1352His	het	25		7	117304834	0.923	23.4	Probably damaging	Deleterious	NA	0.000611	rs113857788	CM931158	NA	VCV000007237.37 Conflicting interpretations of pathogenicity Pathogenic(2); Uncertain significance(10); Benign(1); Likely benign(2)	P	PS3,PS4_Moderate,PM2_Supporting,PM3,PP3_Moderate
AV129Q	37		WT	WT				7													

AV130	27	CBAVD	c.1210_12T[5] (c.1210-7_1210-6del)		het		9	7	117188683	NA	NA	NA	Deleterious	NA	NA	rs1805177	CX931174	Varying clinical consequence	VCV000242535.51 Pathogenic(13); Likely pathogenic(1); Uncertain significance(1); Likely benign(1)	P	PVS1_Strength,PM2_Supporting,PM3
AV130Q	24		WT	WT				7													
AV131	27	CBAVD	c.1210_12T[5] (c.1210-7_1210-6del)		het		9	7	117188683	NA	NA	NA	Deleterious	NA	NA	rs1805177	CX931174	Varying clinical consequence	VCV000242535.51 Pathogenic(13); Likely pathogenic(1); Uncertain significance(1); Likely benign(1)	P	PVS1_Strength,PM2_Supporting,PM3
AV131	27	CBAVD	c.-34C>T		het		5'-UTR	7	117120115	NA	15.48	NA	Deleterious	NA	0.000003988	rs756314710	CR118489	NA	VCV000552672.8 (Pathogenic(1); Uncertain significance(2))	LP	PS3,PM2_Supporting,PM3
AV131Q	27		WT	WT				7													
AV132	33	CBAVD	c.1666A>G	p.Ile556Val	het	12		7	117227874	0.440	19.69	Benign	Deleterious	NA	0.00223	rs75789129	CM920985	NA	VCV000007196.45 (Pathogenic(1); Uncertain significance(1); Benign(4); Likely benign(6))	LB	PS3_Supporting, PM2_Supporting, BS1, BP1
AV132Q	32		WT	WT				7													
AV133	22	CBAVD	c.1210_12T[5] (c.1210-7_1210-6del)		het		9	7	117188683	NA	NA	NA	Deleterious	NA	NA	rs1805177	CX931174	Varying clinical consequence	VCV000242535.51 Pathogenic(13); Likely pathogenic(1); Uncertain significance(1); Likely benign(1)	P	PVS1_Strength,PM2_Supporting,PM3
AV133	22	CBAVD	c.2684G>A	p.Ser895Asn	het	17		7	117243612	0.351	5.990	Benign	Polymorphis	NA	0.0003255	rs201864483	CM004880	NA	VCV000053543.23 Uncertain significance(7); Benign(1); Likely benign(3)	VUS	PM2_Supporting,BP4
AV133Q	25		WT	WT				7													
AV134	34	CUAVD	c.1210_12T[5] (c.1210-7_1210-6del)		het		9	7	117188683	NA	NA	NA	Deleterious	NA	NA	rs1805177	CX931174	Varying clinical consequence	VCV000242535.51 Pathogenic(13); Likely pathogenic(1); Uncertain significance(1); Likely benign(1)	P	PVS1_Strength,PM2_Supporting,PM3
AV134Q	32		WT	WT				7													
AV135	33	CBAVD	c.1210_12T[5] (c.1210-7_1210-6del)		het		9	7	117188683	NA	NA	NA	Deleterious	NA	NA	rs1805177	CX931174	Varying clinical consequence	VCV000242535.51 Pathogenic(13); Likely pathogenic(1); Uncertain significance(1); Likely benign(1)	P	PVS1_Strength,PM2_Supporting,PM3
AV135	33	CBAVD	c.263T>G	p.Leu88*	het	3		7	117149186	NA	37	NA	Deleterious	NA	NA	rs397508412	CM920142	CF-causing	VCV000053534.12 （Pathogenic ）	P	PVS1_Strength,PM2_Supporting,PP3
AV135Q	30		WT	WT				7													
AV136	33	CBAVD	WT	WT				7													
AV136Q	28		WT	WT				7													
AV137	31	CBAVD	c.1210_12T[5] (c.1210-7_1210-6del)		comhet		9	7	117188683	NA	NA	NA	Deleterious	NA	NA	rs1805177	CX931174	Varying clinical consequence	VCV000242535.51 Pathogenic(13); Likely pathogenic(1); Uncertain significance(1); Likely benign(1)	P	PVS1_Strength,PM2_Supporting,PM3
AV137	31	CBAVD	c.1657C>T	p.Arg553*	comhet	12		7	117227865	NA	39	NA	Deleterious	NA	0.00002241	rs74597325	CM900054	CF-causing	VCV000007122.125 (Pathogenic)	P	PVS1_Strength,PM2_Supporting,PP3
AV137Q	32		c.1666A>G	p.Ile556Val	hethet	12		7	117227874	0.440	19.69	Benign	Deleterious	NA	0.00223	rs75789129	CM920985	NA	VCV000007196.45 (Pathogenic(1); Uncertain significance(1); Benign(4); Likely benign(6))	LB	PS3_Supporting, PM2_Supporting, BS1, BP1
AV137Q	32		c.1680-1G>A		hethet			7	117230406	NA	35	NA	Deleterious	0.92	3.996E-06	rs121908794	CS941442	CF-causing	VCV000048681.24 (Pathogenic)	P	PVS1_Strength,PM2_Supporting,PP3
AV138	29	CBAVD	c.1210_12T[5] (c.1210-7_1210-6del)		het		9	7	117188683	NA	NA	NA	Deleterious	NA	NA	rs1805177	CX931174	Varying clinical consequence	VCV000242535.51 Pathogenic(13); Likely pathogenic(1); Uncertain significance(1); Likely benign(1)	P	PVS1_Strength,PM2_Supporting,PM3
AV138	29	CBAVD	c.1343T>C	p.Ile448Thr	het	10		7	117188828	0.913	24.2	Possibly damaging	Deleterious	NA	0.000008208	rs748642635	NA	NA	VCV000526015.9 (Uncertain significance)	VUS	PM2_Supporting,PP3_Moderate
AV138Q	29		WT	WT				7													
AV139	26	CBAVD	c.1666A>G	p.Ile556Val	het	12		7	117227874	0.440	19.69	Benign	Deleterious	NA	0.00223	rs75789129	CM920985	NA	VCV000007196.45 (Pathogenic(1); Uncertain significance(1); Benign(4); Likely benign(6))	LB	PS3_Supporting, PM2_Supporting, BS1, BP1
AV139	26	CBAVD	c.3289C>T	p.Arg1097Cys	het	20		7	117251784	0.820	33	Probably damaging	Deleterious	NA	0.0002691	rs201591901	CM1923248	NA	VCV000574132.15 (Uncertain significance(3); Likely benign(1))	VUS	PM2_Supporting,PP3_Moderate
AV139Q	29		NA	NA				7													
AV140	34	CBAVD	c.1210_12T[5] (c.1210-7_1210-6del)		het		9	7	117188683	NA	NA	NA	Deleterious	NA	NA	rs1805177	CX931174	Varying clinical consequence	VCV000242535.51 Pathogenic(13); Likely pathogenic(1); Uncertain significance(1); Likely benign(1)	P	PVS1_Strength,PM2_Supporting,PM3
AV140	34	CBAVD	c.1666A>G	p.Ile556Val	het	12		7	117227874	0.440	19.69	Benign	Deleterious	NA	0.00223	rs75789129	CM920985	NA	VCV000007196.45 (Pathogenic(1); Uncertain significance(1); Benign(4); Likely benign(6))	LB	PS3_Supporting, PM2_Supporting, BS1, BP1
AV140Q	31		WT	WT				7													
AV141	30	CBAVD	WT	WT				7													
AV141Q	23		WT	WT				7													
AV142	36	CBAVD	c.1210_12T[5] (c.1210-7_1210-6del)		het		9	7	117188683	NA	NA	NA	Deleterious	NA	NA	rs1805177	CX931174	Varying clinical consequence	VCV000242535.51 Pathogenic(13); Likely pathogenic(1); Uncertain significance(1); Likely benign(1)	P	PVS1_Strength,PM2_Supporting,PM3
AV142	36		c.-34C>T		het		5'-UTR	7	117120115	NA	15.48	NA	Deleterious	NA	0.000003988	rs756314710	CR118489	NA	VCV000552672.8 (Pathogenic(1); Uncertain significance(2))	LP	PS3,PM2_Supporting,PM3
AV142Q	36		NA	NA				7													
AV143	36	CBAVD	WT	WT				7													
AV143Q	35		c.1666A>G	p.Ile556Val	het	12		7	117227874	0.440	19.69	Benign	Deleterious	NA	0.00223	rs75789129	CM920985	NA	VCV000007196.45 (Pathogenic(1); Uncertain significance(1); Benign(4); Likely benign(6))	LB	PS3_Supporting, PM2_Supporting, BS1, BP1
AV144	27	CBAVD	c.374T>C	p.Ile125Thr	het	4		7	117171053	0.668	22.2	Probably damaging	Deleterious	NA	0.0006978	rs141723617	CM983538	NA	VCV000053802.32 (Uncertain significance(6); Likely benign(4))	VUS	PM2_Supporting,PM3,PP3
AV144	27	CBAVD	c.959delCinsTT	p.Pro320Leufs*4	hemi	16		X	19031944	NA	NA	NA	Deleterious	NA	NA	NA	NA	NA	NA	P	PVS1,PM2_Supporting,PP3
AV144Q	25		WT	WT				7													
AV145	31	CBAVD	c.1210_12T[5] (c.1210-7_1210-6del)		het		9	7	117188683	NA	NA	NA	Deleterious	NA	NA	rs1805177	CX931174	Varying clinical consequence	VCV000242535.51 Pathogenic(13); Likely pathogenic(1); Uncertain significance(1); Likely benign(1)	P	PVS1_Strength,PM2_Supporting,PM3
AV145	31	CBAVD	c.3139G>T	p.Gly1047Cys	het	19		7	117250723	0.589	34	Possibly damaging	Deleterious	0.96	NA	rs397508504	NA	NA	VCV000283775.15 (Uncertain significance)	P	PVS1_Strength,PM2_Supporting,PP3
AV145Q	31		c.1666A>G	p.Ile556Val	het	12		7	117227874	0.440	19.69	Benign	Deleterious	NA	0.00223	rs75789129	CM920985	NA	VCV000007196.45 (Pathogenic(1); Uncertain significance(1); Benign(4); Likely benign(6))	LB	PS3_Supporting, PM2_Supporting, BS1, BP1
AV146	35	CUAVD	c.1210_12T[5] (c.1210-7_1210-6del)		het		9	7	117188683	NA	NA	NA	Deleterious	NA	NA	rs1805177	CX931174	Varying clinical consequence	VCV000242535.51 Pathogenic(13); Likely pathogenic(1); Uncertain significance(1); Likely benign(1)	P	PVS1_Strength,PM2_Supporting,PM3
AV146	35	CUAVD	c.4056G>C	p.Gln1352His	het	25		7	117304834	0.923	23.4	Probably damaging	Deleterious	NA	0.000611	rs113857788	CM931158	NA	VCV000007237.37 Conflicting interpretations of pathogenicity Pathogenic(2); Uncertain significance(10); Benign(1); Likely benign(2)	P	PS3,PS4_Moderate,PM2_Supporting,PM3,PP3_Moderate
AV146Q	35		WT	WT				7													
AV147	34	CBAVD	c.1210_12T[5] (c.1210-7_1210-6del)		het		9	7	117188683	NA	NA	NA	Deleterious	NA	NA	rs1805177	CX931174	Varying clinical consequence	VCV000242535.51 Pathogenic(13); Likely pathogenic(1); Uncertain significance(1); Likely benign(1)	P	PVS1_Strength,PM2_Supporting,PM3
AV147	34	CBAVD	c.1000C>T	p.Arg334Trp	het	8		7	117180284	0.816	25.3	Possibly damaging	Deleterious	NA	0.00005662	NA	CM910070	CF-causing	NA	P	PM2_Supporting,PP3
AV147Q	25		WT	WT				7													
AV148	31	CBAVD	c.2929T>C	p.Ser977Pro	comhet	18		7	117246748	0.867	25.4	Possibly damaging	Deleterious	NA	0.000003982	rs137975784	CM960285	NA	VCV000053599.2 (Likely pathogenic)	VUS	PM2_Supporting,PM5,PP3_Moderate
AV148	31	CBAVD	c.3469-3C>A		comhet		21	7	117267573	NA	16.98	NA	Deleterious	0.19	0.00003994	NA	CS158229	NA	VCV001731705.5 (Uncertain significance)	LB	PM2_Supporting,PM3,PP3,BS3,BP4
AV148Q	30		c.1666A>G	p.Ile556Val	het	12		7	117227874	0.440	19.69	Benign	Deleterious	NA	0.00223	rs75789129	CM920985	NA	VCV000007196.45 (Pathogenic(1); Uncertain significance(1); Benign(4); Likely benign(6))	LB	PS3_Supporting, PM2_Supporting, BS1, BP1
AV149	33	CBAVD	c.1210_12T[5] (c.1210-7_1210-6del)		het		9	7	117188683	NA	NA	NA	Deleterious	0.05	NA	rs1805177	CX931174	Varying clinical consequence	VCV000242535.51 Pathogenic(13); Likely pathogenic(1); Uncertain significance(1); Likely benign(1)	P	PVS1_Strength,PM2_Supporting,PM3
AV149	33	CBAVD	c.4433C>G	p.Thr1478Arg	het	27		7	117307152	0.600	25.1	Probably damaging	Deleterious	NA	0.00001595	rs753173837	CM158227	NA	VCV000824887.7(Uncertain significance)	VUS	PM2_Supporting,PP3
AV149Q	31		WT	WT				7													
AV150	33	CBAVD	c.4056G>C	p.Gln1352His	het	25		7	117304834	0.923	23.4	Probably damaging	Deleterious	NA	0.000611	rs113857788	CM931158	NA	VCV000007237.37 Conflicting interpretations of pathogenicity Pathogenic(2); Uncertain significance(10); Benign(1); Likely benign(2)	P	PS3,PS4_Moderate,PM2_Supporting,PM3,PP3_Moderate
AV150Q	31		WT	WT				7													
AV151	30	CBAVD	WT	WT				7													
AV151Q	30		c.1210_12T[6] (c.1210-6delT)		het		9	7	117188683	NA	NA	NA	Benign	0.02	NA	NA	NA	NA	VCV000495892.8 (Uncertain significance)	VUS	PM2_Supporting,BP4
AV152	31	CBAVD	c.1210_12T[5] (c.1210-7_1210-6del)		het		9	7	117188683	NA	NA	NA	Deleterious	0.05	NA	rs1805177	CX931174	Varying clinical consequence	VCV000242535.51 Pathogenic(13); Likely pathogenic(1); Uncertain significance(1); Likely benign(1)	P	PVS1_Strength,PM2_Supporting,PM3
AV152Q	27		c.3659C>T	p.Thr1220Ile	het	22		7	117267766	0.400	0.219	Benign	polymorphis	NA	0.00009945	rs1800123	CD972131	CF-causing	VCV000007214.24 (Uncertain significance)	P	PM2_Supporting
AV153	30	CBAVD	c.1210_12T[5] (c.1210-7_1210-6del)		het		9	7	117188683	NA	NA	NA	Deleterious	0.05	NA	rs1805177	CX931174	Varying clinical consequence	VCV000242535.51 Pathogenic(13); Likely pathogenic(1); Uncertain significance(1); Likely benign(1)	P	PVS1_Strength,PM2_Supporting,PM3
AV153	30	CBAVD	c.1438G>A	p.Gly480Ser	het	11		7	117199563	0.992	28.8	Probably damaging	Deleterious	NA	NA	rs79282516	CM015355	NA	VCV000053254.15(Likely pathogenic(1); Uncertain significance(6))	LP	PM2_Supporting,PM5,PP3_Strong
AV153Q	26		WT	WT				7													
AV155	31	CUAVD	c.1210_12T[5] (c.1210-7_1210-6del)		het		9	7	117188683	NA	NA	NA	Deleterious	0.05	NA	rs1805177	CX931174	Varying clinical consequence	VCV000242535.51 Pathogenic(13); Likely pathogenic(1); Uncertain significance(1); Likely benign(1)	P	PVS1_Strength,PM2_Supporting,PM3
AV155	31	CUAVD	c.1666A>G	p.Ile556Val	het	12		7	117227874	0.440	19.69	Benign	Deleterious	NA	0.00223	rs75789129	CM920985	NA	VCV000007196.45 (Pathogenic(1); Uncertain significance(1); Benign(4); Likely benign(6))	LB	PS3_Supporting, PM2_Supporting, BS1, BP1
AV155Q	31		NA	NA				7													
AV156	34	CUAVD	c.4297G>A	p.Glu1433Lys	het	27		7	117307016	0.602	26.1	Probably damaging	Deleterious	NA	2.392E-05	rs750559671	CM176228	NA	VCV000558250.11(Uncertain significance)	VUS	PM2_Supporting,PP3
AV156Q	25		WT	WT				7													
AV157	25	CUAVD	c.1210_12T[5] (c.1210-7_1210-6del)		comhet		9	7	117188683	NA	NA	NA	Deleterious	0.05	NA	rs1805177	CX931174	Varying clinical consequence	VCV000242535.51 Pathogenic(13); Likely pathogenic(1); Uncertain significance(1); Likely benign(1)	P	PVS1_Strength,PM2_Supporting,PM3
AV157	25	CUAVD	c.374T>C	p.Ile125Thr	comhet	4		7	117171053	0.668	22.2	Probably damaging	Deleterious	NA	0.0006978	rs141723617	CM983538	NA	VCV000053802.32 (Uncertain significance(6); Likely benign(4))	VUS	PM2_Supporting,PM3,PP3
AV157Q	25		c.1666A>G	p.Ile556Val	het	12		7	117227874	0.440	19.69	Benign	Deleterious	NA	0.00223	rs75789129	CM920985	NA	VCV000007196.45 (Pathogenic(1); Uncertain significance(1); Benign(4); Likely benign(6))	LB	PS3_Supporting, PM2_Supporting, BS1, BP1
AV158	38	CBAVD	c.1210_12T[5] (c.1210-7_1210-6del)		homo		9	7	117188683	NA	NA	NA	Deleterious	0.05	NA	rs1805177	CX931174	Varying clinical consequence	VCV000242535.51 Pathogenic(13); Likely pathogenic(1); Uncertain significance(1); Likely benign(1)	P	PVS1_Strength,PM2_Supporting,PM3
AV158Q	33		WT	WT				7													
AV159	33	CUAVD	c.1210_12T[5] (c.1210-7_1210-6del)		het		9	7	117188683	NA	NA	NA	Deleterious	0.05	NA	rs1805177	CX931174	Varying clinical consequence	VCV000242535.51 Pathogenic(13); Likely pathogenic(1); Uncertain significance(1); Likely benign(1)	P	PVS1_Strength,PM2_Supporting,PM3
AV159	33	CUAVD	c.2249C>T	p.Pro750Leu	het	14		7	117232470	0.655	17.07	Benign	Deleterious	NA	0.0002843	rs140455771	CM000667	CF-causing	VCV000053460.44 (Pathogenic(1); Likely pathogenic(17); Uncertain significance(5))	P	PS3,PM2_Supporting,PP3
AV159Q	34		c.3205G>A	p.Gly1069Arg	het	20		7	117251700	0.603	23.5	Probably damaging	Deleterious	NA	0.00009556	rs200321110	CM003252	CF-causing	VCV000053684.40 (Pathogenic(2); Likely pathogenic(4); Uncertain significance(8))	P	PS3,PM2_Supporting,PM3,PP3
AV159Q	34		c.4056G>C	p.Gln1352His	het	25		7	117304834	0.923	23.4	Probably damaging	Deleterious	NA	0.000611	rs113857788	CM931158	NA	VCV000007237.37 Conflicting interpretations of pathogenicity Pathogenic(2); Uncertain significance(10); Benign(1); Likely benign(2)	P	PS3,PS4_Moderate,PM2_Supporting,PM3,PP3_Moderate
AV160	29	CUAVD	c.1210_12T[5] (c.1210-7_1210-6del)		comhet		9	7	117188683	NA	NA	NA	Deleterious	0.05	NA	rs1805177	CX931174	Varying clinical consequence	VCV000242535.51 Pathogenic(13); Likely pathogenic(1); Uncertain significance(1); Likely benign(1)	P	PVS1_Strength,PM2_Supporting,PM3
AV160	29	CUAVD	c.4056G>C	p.Gln1352His	comhet	25		7	117304834	0.923	23.4	Probably damaging	Deleterious	NA	0.000611	rs113857788	CM931158	NA	VCV000007237.37 Conflicting interpretations of pathogenicity Pathogenic(2); Uncertain significance(10); Benign(1); Likely benign(2)	P	PS3,PS4_Moderate,PM2_Supporting,PM3,PP3_Moderate
AV160Q	29		NA	NA				7													
AV161	31	CBAVD	WT	WT				7													
AV161Q	29		WT	WT				7													
AV162	25	CBAVD	WT	WT				7													
AV162Q	26		WT	WT				7													
AV163	29	CBAVD	c.1210_12T[5] (c.1210-7_1210-6del)		homo		9	7	117188683	NA	NA	NA	Deleterious	0.05	NA	rs1805177	CX931174	Varying clinical consequence	VCV000242535.51 Pathogenic(13); Likely pathogenic(1); Uncertain significance(1); Likely benign(1)	P	PVS1_Strength,PM2_Supporting,PM3
AV163Q	28		WT	WT				7													
AV164	28	CBAVD	WT	WT				7													
AV164Q	28		c.1666A>G	p.Ile556Val	het	12		7	117227874	0.440	19.69	Benign	Deleterious	NA	0.00223	rs75789129	CM920985	NA	VCV000007196.45 (Pathogenic(1); Uncertain significance(1); Benign(4); Likely benign(6))	LB	PS3_Supporting, PM2_Supporting, BS1, BP1
AV165	35	CBAVD	c.1210_12T[5] (c.1210-7_1210-6del)		homo		9	7	117188683	NA	NA	NA	Deleterious	0.05	NA	rs1805177	CX931174	Varying clinical consequence	VCV000242535.51 Pathogenic(13); Likely pathogenic(1); Uncertain significance(1); Likely benign(1)	P	PVS1_Strength,PM2_Supporting,PM3
AV165Q	35		NA	NA				7													
AV166	31	CUAVD	c.1210_12T[5] (c.1210-7_1210-6del)		het		9	7	117188683	NA	NA	NA	Deleterious	0.05	NA	rs1805177	CX931174	Varying clinical consequence	VCV000242535.51 Pathogenic(13); Likely pathogenic(1); Uncertain significance(1); Likely benign(1)	P	PVS1_Strength,PM2_Supporting,PM3
AV166	31	CUAVD	c.-34C>T		het		5'-UTR	7	117120115	NA	15.48	NA	Deleterious	NA	0.000003988	rs756314710	CR118489	NA	VCV000552672.8 (Pathogenic(1); Uncertain significance(2))	LP	PS3,PM2_Supporting,PM3
AV166Q	31		WT	WT				7													
AV167	30	CBAVD	c.1210_12T[5] (c.1210-7_1210-6del)		het		9	7	117188683	NA	NA	NA	Deleterious	0.05	NA	rs1805177	CX931174	Varying clinical consequence	VCV000242535.51 Pathogenic(13); Likely pathogenic(1); Uncertain significance(1); Likely benign(1)	P	PVS1_Strength,PM2_Supporting,PM3
AV167	30	CBAVD	c.2909G>A	p.Gly970Asp	het	18		7	117246728	0.985	32	Probably damaging	Deleterious	0.32	0.0002	rs386134230	CM990363	CF-causing	VCV000035854.26 (Pathogenic)	P	PVS1_Strength,PS3,PM2_Supporting,PM3,PP3
AV167Q	30		WT	WT				7													
AV168	31	CBAVD	c.1210_12T[5] (c.1210-7_1210-6del)		het		9	7	117188683	NA	NA	NA	Deleterious	0.05	NA	rs1805177	CX931174	Varying clinical consequence	VCV000242535.51 Pathogenic(13); Likely pathogenic(1); Uncertain significance(1); Likely benign(1)	P	PVS1_Strength,PM2_Supporting,PM3
AV168	31	CBAVD	c.3205G>A	p.Gly1069Arg	het	20		7	117251700	0.603	23.5	Probably damaging	Deleterious	NA	0.00009556	rs200321110	CM003252	CF-causing	VCV000053684.40 (Pathogenic(2); Likely pathogenic(4); Uncertain significance(8))	P	PS3,PM2_Supporting,PM3,PP3
AV168Q	29		c.2552G>A	p.Arg851Gln	het	15		7	117235045	0.799	29.3	Probably damaging	Deleterious	NA	0.00009907	rs397508395	NA	NA	VCV000411119.20(Uncertain significance)	VUS	PM2_Supporting,PP3_Moderate
AV169	33	CBAVD	c.1210_12T[5] (c.1210-7_1210-6del)		het		9	7	117188683	NA	NA	NA	Deleterious	0.05	NA	rs1805177	CX931174	Varying clinical consequence	VCV000242535.51 Pathogenic(13); Likely pathogenic(1); Uncertain significance(1); Likely benign(1)	P	PVS1_Strength,PM2_Supporting,PM3
AV169	33	CBAVD	c.523A>G	p.Ile175Val	het	5		7	117174363	0.752	33	Probably damaging	Deleterious	NA	NA	rs397508744	CM940244	NA	VCV000053986.12(Pathogenic(2); Likely pathogenic(2); Uncertain significance(3))	VUS	PM2_Supporting,PP3
AV169Q	31		NA	NA				7													
AV170	30	CUAVD	c.4056G>C	p.Gln1352His	het	25		7	117304834	0.923	23.4	Probably damaging	Deleterious	NA	0.000611	rs113857788	CM931158	NA	VCV000007237.37 Conflicting interpretations of pathogenicity Pathogenic(2); Uncertain significance(10); Benign(1); Likely benign(2)	P	PS3,PS4_Moderate,PM2_Supporting,PM3,PP3_Moderate
AV170Q	26		c.3289C>T	p.Arg1097Cys	het	20		7	117251784	0.820	33	Probably damaging	Deleterious	NA	0.0002691	rs201591901	CM1923248	NA	VCV000574132.15 (Uncertain significance(3); Likely benign(1))	VUS	PM2_Supporting,PP3_Moderate
AV171	35	CBAVD	WT	WT				7													
AV171Q	29		WT	WT				7													
AV172	35	CUAVD	WT	WT				7													

AV172Q	29		WT	WT				7													
AV173	33	CBAVD	c.1666A>G	p.Ile556Val	het	12		7	117227874	0.440	19.69	Benign	Deleterious	NA	0.00223	rs75789129	CM920985	NA	VCV000007196.45 (Pathogenic(1); Uncertain significance(1); Benign(4); Likely benign(6))	LB	PS3_Supporting, PM2_Supporting, BS1, BP1
AV173Q	32		WT	WT				7													
AV174	32	CBAVD	c.1210_12T[5] (c.1210-7_1210-6del)		het		9	7	117188683	NA	NA	NA	Deleterious	0.05	NA	rs1805177	CX931174	Varying clinical consequence	VCV000242535.51 Pathogenic(13); Likely pathogenic(1); Uncertain significance(1); Likely benign(1)	P	PVS1_Strength,PM2_Supporting,PM3
AV174	32	CBAVD	c.4056G>C	p.Gln1352His	het	25		7	117304834	0.923	23.4	Probably damaging	Deleterious	NA	0.000611	rs113857788	CM931158	NA	VCV000007237.37 Conflicting interpretations of pathogenicity Pathogenic(2); Uncertain significance(10); Benign(1); Likely benign(2)	P	PS3,PS4_Moderate,PM2_Supporting,PM3,PP3_Moderate
AV174Q	28		c.1951G>A	p.Asp651Asn	het	14		7	117232172	0.791	31	Probably damaging	Deleterious	NA	0.00003188	rs780526529	CM983561	NA	VCV000820398.9 (Uncertain significance)	VUS	PM2_Supporting,PP3_Moderate
AV174Q	28		c.4056G>C	p.Gln1352His	het	25		7	117304834	0.923	23.4	Probably damaging	Deleterious	NA	0.000611	rs113857788	CM931158	NA	VCV000007237.37 Conflicting interpretations of pathogenicity Pathogenic(2); Uncertain significance(10); Benign(1); Likely benign(2)	P	PS3,PS4_Moderate,PM2_Supporting,PM3,PP3_Moderate
AV175	34	CBAVD	c.1210_12T[5] (c.1210-7_1210-6del)		het		9	7	117188683	NA	NA	NA	Deleterious	0.05	NA	rs1805177	CX931174	Varying clinical consequence	VCV000242535.51 Pathogenic(13); Likely pathogenic(1); Uncertain significance(1); Likely benign(1)	P	PVS1_Strength,PM2_Supporting,PM3
AV175	34	CBAVD	c.1585-2A>G		het		11	7	117227791	NA	33	NA	Deleterious	0.97	NA	rs397508233	CS962493	CF-causing	VCV000053290.4 (Pathogenic/Likely pathogenic)	P	PVS1_Strength,PM2_Supporting,PP3
AV175Q	38		WT	WT				7													
AV176	29	CBAVD	WT	WT				7													
AV176Q	24		WT	WT				7													
AV177	29	CBAVD	c.1210_12T[5] (c.1210-7_1210-6del)		het		9	7	117188683	NA	NA	NA	Deleterious	0.05	NA	rs1805177	CX931174	Varying clinical consequence	VCV000242535.51 Pathogenic(13); Likely pathogenic(1); Uncertain significance(1); Likely benign(1)	P	PVS1_Strength,PM2_Supporting,PM3
AV177	29	CBAVD	c.3469-3C>A		het		21	7	117267573	NA	16.98	NA	Deleterious	0.19	0.00003994	NA	CS158229	NA	VCV001731705.5(Uncertain significance)	LB	PM2_Supporting,PP3,BS3,BP4
AV177Q	31		WT	WT				7													
AV178	29	CBAVD	c.263T>G	p.Leu88*	het	3		7	117149186	NA	37	NA	Deleterious	NA	NA	rs397508412	CM920142	CF-causing	VCV000053534.12 （Pathogenic ）	P	PVS1_Strength,PM2_Supporting,PP3
AV178	29	CBAVD	c.4056G>C	p.Gln1352His	het	25		7	117304834	0.923	23.4	Probably damaging	Deleterious	NA	0.000611	rs113857788	CM931158	NA	VCV000007237.37 Conflicting interpretations of pathogenicity Pathogenic(2); Uncertain significance(10); Benign(1); Likely benign(2)	P	PS3,PS4_Moderate,PM2_Supporting,PM3,PP3_Moderate
AV178Q	29		WT	WT				7													
AV179	37	CBAVD	c.1210_12T[5] (c.1210-7_1210-6del)		homo		9	7	117188683	NA	NA	NA	Deleterious	0.05	NA	rs1805177	CX931174	Varying clinical consequence	VCV000242535.51 Pathogenic(13); Likely pathogenic(1); Uncertain significance(1); Likely benign(1)	P	PVS1_Strength,PM2_Supporting,PM3
AV179Q	30		WT	WT				7													
AV180	26	CBAVD	c.1210_12T[5] (c.1210-7_1210-6del)		homo		9	7	117188683	NA	NA	NA	Deleterious	0.05	NA	rs1805177	CX931174	Varying clinical consequence	VCV000242535.51 Pathogenic(13); Likely pathogenic(1); Uncertain significance(1); Likely benign(1)	P	PVS1_Strength,PM2_Supporting,PM3
AV180Q	26		WT	WT				7													
AV181	33	CBAVD	WT	WT				7													
AV181Q	33		WT	WT				7											VCV000242535.51 Pathogenic(13); Likely pathogenic(1); Uncertain significance(1); Likely benign(1)		
AV182	32	CBAVD	c.1210_12T[5] (c.1210-7_1210-6del)		het		9	7	117188683	NA	NA	NA	Deleterious	0.05	NA	rs1805177	CX931174	Varying clinical consequence		P	PVS1_Strength,PM2_Supporting,PM3
AV182	32	CBAVD	c.3205G>A	p.Gly1069Arg	het	20		7	117251700	0.603	23.5	Probably damaging	Deleterious	NA	0.00009556	rs200321110	CM003252	CF-causing	VCV000053684.40 (Pathogenic(2); Likely pathogenic(4); Uncertain significance(8))	P	PS3,PM2_Supporting,PM3,PP3
AV182Q	26		WT	WT				7													
AV183	29	CBAVD	WT	WT				7													
AV183Q	29		WT	WT				7													
AV184	32	CBAVD	WT	WT				7													
AV184Q	33		WT	WT				7													
AV185	30	CBAVD	c.1210_12T[5] (c.1210-7_1210-6del)		het		9	7	117188683	NA	NA	NA	Deleterious	0.05	NA	rs1805177	CX931174	Varying clinical consequence	VCV000242535.51 Pathogenic(13); Likely pathogenic(1); Uncertain significance(1); Likely benign(1)	P	PVS1_Strength,PM2_Supporting,PM3
AV185	30	CBAVD	c.2936A>C	p.Asp979Ala	het	18		7	117246755	0.990	28.0	Probably damaging	Deleterious	NA	0.00003583	rs397508462	CM970291	CF-causing	VCV000053602.56 (Likely pathogenic(3); Uncertain significance(3))	P	PS3,PM2_Supporting,PM5,PP3
AV185Q	30		c.2904A>C	p.Lys968Asn	het	18		7	117243832	0.584	24.7	Probably damaging	Deleterious	NA	NA	NA	NA	NA	NA	VUS	PM1,PM2_Supporting,PP3
AV186	45	CBAVD	c.1210_12T[5] (c.1210-7_1210-6del)		het		9	7	117188683	NA	NA	NA	Deleterious	0.05	NA	rs1805177	CX931174	Varying clinical consequence	VCV000242535.51 Pathogenic(13); Likely pathogenic(1); Uncertain significance(1); Likely benign(1)	P	PVS1_Strength,PM2_Supporting,PM3
AV186Q	37		c.1666A>G	p.Ile556Val	het	12		7	117227874	0.440	19.69	Benign	Deleterious	NA	0.00223	rs75789129	CM920985	NA	VCV000007196.45 (Pathogenic(1); Uncertain significance(1); Benign(4); Likely benign(6))	LB	PS3_Supporting, PM2_Supporting, BS1, BP1
AV187	31	CBAVD	c.1766+5G>T		comhet		13	7	117230498	NA	20.7	NA	Deleterious	0.69	0.00006	rs121908796	CS951375	CF-causing	VCV000048685.25 (Pathogenic)	P	PVS1_Strength,PM2_Supporting,PP3
AV187	31	CBAVD	c.4056G>C	p.Gln1352His	comhet	25		7	117304834	0.923	23.4	Probably damaging	Deleterious	NA	0.000611	rs113857788	CM931158	NA	VCV000007237.37 Conflicting interpretations of pathogenicity Pathogenic(2); Uncertain significance(10); Benign(1); Likely benign(2)	P	PS3,PS4_Moderate,PM2_Supporting,PM3,PP3_Moderate
AV187Q	31		c.1666A>G	p.Ile556Val	het	12		7	117227874	0.440	19.69	Benign	Deleterious	NA	0.00223	rs75789129	CM920985	NA	VCV000007196.45 (Pathogenic(1); Uncertain significance(1); Benign(4); Likely benign(6))	LB	PS3_Supporting, PM2_Supporting, BS1, BP1
AV188	28	CBAVD	c.373dupA	p.Ile125Asnfs*34	comhet	4		7	117171052	NA	NA	NA	Deleterious	NA	NA	NA	NA	NA	NA	P	PVS1,PM2_Supporting,PM3
AV188	28	CBAVD	c.4056G>C	p.Gln1352His	comhet	25		7	117304834	0.923	23.4	Probably damaging	Deleterious	NA	0.000611	rs113857788	CM931158	NA	VCV000007237.37 Conflicting interpretations of pathogenicity Pathogenic(2); Uncertain significance(10); Benign(1); Likely benign(2)	P	PS3,PS4_Moderate,PM2_Supporting,PM3,PP3_Moderate
AV188Q	25		WT	WT				7													
AV189	28	CBAVD	WT	WT				7													
AV189Q	28		WT	WT				7													
AV190	35	CBAVD	c.1210_12T[5] (c.1210-7_1210-6del)		homo		9	7	117188683	NA	NA	NA	Deleterious	0.05	NA	rs1805177	CX931174	Varying clinical consequence	VCV000242535.51 Pathogenic(13); Likely pathogenic(1); Uncertain significance(1); Likely benign(1)	P	PVS1_Strength,PM2_Supporting,PM3
AV190Q	31		c.1666A>G	p.Ile556Val	comhet	12		7	117227874	0.440	19.69	Benign	Deleterious	NA	0.00223	rs75789129	CM920985	NA	VCV000007196.45 (Pathogenic(1); Uncertain significance(1); Benign(4); Likely benign(6))	LB	PS3_Supporting, PM2_Supporting, BS1, BP1
AV190Q	31		c.2908+4C>A		comhet		17	7	117243840	NA	11.78	NA	Benign	0.09	0.00003587	rs372266030	NA	NA	VCV000596852.8 (Uncertain significance)	LB	PM2_Supporting,BS3,BP4
AV191	26	CBAVD	c.1210_12T[5] (c.1210-7_1210-6del)		het		9	7	117188683	NA	NA	NA	Deleterious	0.05	NA	rs1805177	CX931174	Varying clinical consequence	VCV000242535.51 Pathogenic(13); Likely pathogenic(1); Uncertain significance(1); Likely benign(1)	P	PVS1_Strength,PM2_Supporting,PM3
AV191	26	CBAVD	c.1342delA	p.Ile448*	het	10		7	117188827	NA	NA	NA	Deleterious	NA	NA	NA	NA	NA	NA	P	PVS1,PM2_Supporting,PP3
AV191Q	26		WT	WT				7													
AV192	41	CBAVD	c.1210_12T[5] (c.1210-7_1210-6del)		het		9	7	117188683	NA	NA	NA	Deleterious	0.05	NA	rs1805177	CX931174	Varying clinical consequence	VCV000242535.51 Pathogenic(13); Likely pathogenic(1); Uncertain significance(1); Likely benign(1)	P	PVS1_Strength,PM2_Supporting,PM3
AV192	41	CBAVD	c.1666A>G	p.Ile556Val	het	12		7	117227874	0.440	19.69	Benign	Deleterious	NA	0.00223	rs75789129	CM920985	NA	VCV000007196.45 (Pathogenic(1); Uncertain significance(1); Benign(4); Likely benign(6))	LB	PS3_Supporting, PM2_Supporting, BS1, BP1
AV192	41	CBAVD	c.4056G>C	p.Gln1352His	het	25		7	117304834	0.923	23.4	Probably damaging	Deleterious	NA	0.000611	rs113857788	CM931158	NA	VCV000007237.37 Conflicting interpretations of pathogenicity Pathogenic(2); Uncertain significance(10); Benign(1); Likely benign(2)	P	PS3,PS4_Moderate,PM2_Supporting,PM3,PP3_Moderate
AV192Q	41		WT	WT				7													
AV193	30	CBAVD	WT	WT				7													
AV193Q	30		WT	WT				7													
AV194	37	CBAVD	c.1210_12T[5] (c.1210-7_1210-6del)		het		9	7	117188683	NA	NA	NA	Deleterious	0.05	NA	rs1805177	CX931174	Varying clinical consequence	VCV000242535.51 Pathogenic(13); Likely pathogenic(1); Uncertain significance(1); Likely benign(1)	P	PVS1_Strength,PM2_Supporting,PM3
AV194	37	CBAVD	c.1666A>G	p.Ile556Val	het	12		7	117227874	0.440	19.69	Benign	Deleterious	NA	0.00223	rs75789129	CM920985	NA	VCV000007196.45 (Pathogenic(1); Uncertain significance(1); Benign(4); Likely benign(6))	LB	PS3_Supporting, PM2_Supporting, BS1, BP1
AV194Q	36		WT	WT				7													
AV195	36	CBAVD	WT	WT				7													
AV195Q	32		WT	WT				7													
AV196	37	CBAVD	c.1666A>G	p.Ile556Val	het	12		7	117227874	0.440	19.69	Benign	Deleterious	NA	0.00223	rs75789129	CM920985	NA	VCV000007196.45(Pathogenic(1); Uncertain significance(1); Benign(4); Likely benign(6))	LB	PS3_Supporting, PM2_Supporting, BS1, BP1
AV196Q	32		WT	WT				7													
AV197	31	CBAVD	WT	WT				7													
AV197Q	29		WT	WT				7													
AV198	39	CBAVD	WT	WT				7													
AV198Q	32		NA	NA				7													
AV199	26	CBAVD	c.1210_12T[5] (c.1210-7_1210-6del)		het		9	7	117188683	NA	NA	NA	Deleterious	0.05	NA	rs1805177	CX931174	Varying clinical consequence	VCV000242535.51 Pathogenic(13); Likely pathogenic(1); Uncertain significance(1); Likely benign(1)	P	PVS1_Strength,PM2_Supporting,PM3
AV199	26	CBAVD	c.2812G>T	p.Val938Leu	het	17		7	117243740	0.640	24.7	Probably damaging	Deleterious	NA	0.00002387	rs749784731	NA	NA	VCV000928496.10 (Uncertain significance)	VUS	PM2_Supporting,PP3
AV199	26	CBAVD	c.2907A>C	p.Ala969=	het	17		7	117243835	NA	18.43	NA	Deleterious	0.44	0.00002389	rs377502207	CS165915	CF-causing	VCV000549917.10 (Likely pathogenic(1); Uncertain significance(3); Likely benign(1))	P	PVS1_Strength,PM2_Supporting,PP3
AV199Q	25		c.1666A>G	p.Ile556Val	het	12		7	117227874	0.440	19.69	Benign	Deleterious	NA	0.00223	rs75789129	CM920985	NA	VCV000007196.45 (Pathogenic(1); Uncertain significance(1); Benign(4); Likely benign(6))	LB	PS3_Supporting, PM2_Supporting, BS1, BP1
AV200	43	CBAVD	WT	WT				7													
AV200Q	34		WT	WT				7													
AV201	25	CBAVD	c.433C>G	p.Leu145Val	comhet	4		7	117171112	0.712	24.9	Probably damaging	Deleterious	NA	NA	rs749836021	NA	NA	NA	LP	PM2_Supporting,PM3,PM5,PP3
AV201	25	CBAVD	c.1837G>A	p.Ala613Thr	comhet	14		7	117232058	0.830	26.9	Probably damaging	Deleterious	NA	0.00003186	rs201978662	CM980342	CF-causing	VCV000053401.12 (Pathogenic)	P	
AV201Q	30		WT	WT				7													

Abbreviation: ACMG, The American College of Medical Genetics and Genomics; AF, allele frequency; CADD, Combined Annotation Dependent Depletion; CBVAD, congenital bilateral absence of vas deferens; CUAVD, congenital unilateral absence of the vas deferens; het, heterozygote; hemi, hemizygote; homo, homozygote; comhet, compound heterzygote indicating the variants in trans in a individual; hethet, compound heterzygote indicating the variants in cis in a individual; HGMD, The Human Gene Mutation Database; LB, Likely benign; LP, Likely pathogenic; NA, Not Available; P, pathogenic; REVEL, Rare Exome Variant Ensemble Learner; VUS, variant of uncertain significance; WT, wild type; Sample number with the suffix 'Q' indicates patients' wives.
