# Supplementary material for: Spectrum and Classification of CFTR and ADGRG2 Variants in Chinese Patients With Isolated CAVD: A Large Cohort Study and Risk Assessment of CFTR Variant Carriage in Couples
Source: Hum Mutat. 2026 May 25;2026:5588277. doi: 10.1155/humu/5588277 (PMC13199994; doi:10.1155/humu/5588277)
Supplement: Supplementary file 2 — Supporting Information 2 Table S2: RT‐PCR primers for four variants at the intron‐exon junctions of the CFTR gene. [file HUMU-2026-5588277-s002.docx]

| **Table S2 RT-PCR primiers** | | |
| --- | --- | --- |
| Target gene | Variant | Oligo sequences |
| *CFTR* | c.3469-3C>A | F: GGTATTATCCTGACTTTAGCCAT |
|  |  | R: GTACTCTTCCCTGATCCAGT |
|  | c.579+4T>C | F: TAGCTTCCTATGACCCGGAT |
|  |  | R: GACGCCTGTAACAACTCCC |
|  | c.1210-6T>A | F: AGCAAGAATATAAGACATTGGA |
|  |  | R: ACTGTGCTTAATTTTACCCTC |
|  | c.2908+4C>A | F: GCACCAGTTCGTATTATGTGT |
|  |  | R: TGAAGTCAAATATGGTAAGAGGC |

Abbreviations: F, Forward primer; R, Reverse primer; RT-PCR, Reverse transcription-polymerase chain reaction.
